# Improving Crowdsourcing-Based Image Classification Through Expanded Input Elicitation and Machine Learning

**DOI:** 10.3389/frai.2022.848056

**Published:** 2022-06-29

**Authors:** Romena Yasmin, Md Mahmudulla Hassan, Joshua T. Grassel, Harika Bhogaraju, Adolfo R. Escobedo, Olac Fuentes

**Affiliations:** ^1^School of Computing and Augmented Intelligence, Arizona State University, Tempe, AZ, United States; ^2^Department of Computer Science, University of Texas at El Paso, El Paso, TX, United States

**Keywords:** machine learning, input elicitations, crowdsourcing, human computation, image classification

## Abstract

This work investigates how different forms of input elicitation obtained from crowdsourcing can be utilized to improve the quality of inferred labels for image classification tasks, where an image must be labeled as either positive or negative depending on the presence/absence of a specified object. Five types of input elicitation methods are tested: binary classification (positive or negative); the (*x, y*)-coordinate of the position participants believe a target object is located; level of confidence in binary response (on a scale from 0 to 100%); what participants believe the majority of the other participants' binary classification is; and participant's perceived difficulty level of the task (on a discrete scale). We design two crowdsourcing studies to test the performance of a variety of input elicitation methods and utilize data from over 300 participants. Various existing voting and machine learning (ML) methods are applied to make the best use of these inputs. In an effort to assess their performance on classification tasks of varying difficulty, a systematic synthetic image generation process is developed. Each generated image combines items from the *MPEG-7 Core Experiment CE-Shape-1 Test Set* into a single image using multiple parameters (e.g., density, transparency, etc.) and may or may not contain a target object. The difficulty of these images is validated by the performance of an automated image classification method. Experiment results suggest that more accurate results can be achieved with smaller training datasets when both the crowdsourced binary classification labels and the average of the self-reported confidence values in these labels are used as features for the ML classifiers. Moreover, when a relatively larger properly annotated dataset is available, in some cases augmenting these ML algorithms with the results (i.e., probability of outcome) from an automated classifier can achieve even higher performance than what can be obtained by using any one of the individual classifiers. Lastly, supplementary analysis of the collected data demonstrates that other performance metrics of interest, namely reduced false-negative rates, can be prioritized through special modifications of the proposed aggregation methods.

## 1. Introduction

In recent years, computer vision approaches based on machine learning (ML) and, in particular, those based on deep convolutional neural networks have demonstrated significant performance improvements over conventional approaches for image classification and annotation (Krizhevsky et al., 2012; Tan and Le, 2019; Zhai et al., 2021). However, these algorithms generally require a large and diverse set of annotated data to generate accurate classifications. Large amounts of annotated data are not always available, especially for tasks where producing high-quality meta-data is costly, such as image-based medical diagnosis (Cheplygina et al., 2019), pattern recognition in geospatial remote sensing data (Rasp et al., 2020; Stevens et al., 2020), etc. In addition, ML algorithms are often sensitive to perturbations in the data for complex visual tasks, that to some extent are even difficult for humans, such as object detection in cluttered backgrounds and detection of adversarial examples (McDaniel et al., 2016; Papernot et al., 2016), due to the high dimensionality and variability of the feature space of the images.

Crowdsourcing has received significant attention in various domain-specific applications as a complementary approach for image classification. Its growth has been accompanied and propelled by the emergence of online crowdsourcing platforms (e.g., Amazon Mechanical Turk, Prolific), which are widely employed to recruit and compensate human participants for annotating and classifying data that are difficult for machine-only approaches. In general, crowdsourcing works by leveraging the concept of the “wisdom of the crowd” (Surowiecki, 2005), with which the judgments or predictions of multiple participants are aggregated to sift out noise and to better approximate a ground truth (Yi et al., 2012). Numerous studies over the last decade have established that, under the right circumstances and with the proper aggregation methods, the collective judgment of multiple non-experts is uncontroversially more accurate than those from almost any individual, including well-informed experts. This concept of using groups to make collective decisions has been successfully applied to a number of visual tasks ranging from simple classification and annotation (Russakovsky et al., 2015) to complex real-world applications, including assessment of damages caused by natural disasters (Barrington et al., 2012) and segmentation of biomedical images for diagnostic purposes (Gurari et al., 2015).

Although ML methods have been shown to perform exceedingly well in various classification tasks, these outcomes typically depend on relatively large datasets (Hsing et al., 2018). However, high amounts of richly annotated data are inaccessible in various situations and/or obtaining them is prohibitively costly. Yet in such situations where less data is available, ML methods provide a natural mechanism for incorporating multiple forms of crowdsourced inputs, since they are tailor-made for classification based on input features. Previous works have tended to use a single form of input (i.e., mostly binary classification labels provided by participants) as a feature for ML algorithms on visual classification tasks. However, the vast majority have overlooked other inputs that can be elicitated from the crowd. Formal studies on the merits and potential impacts of different types of elicited inputs are also lacking. This work investigates how the performance of crowdsourcing-based voting and ML methods for image classification tasks can be improved using a variety of inputs. In summary, the contributions of this work stem from the following objectives:

- Analyze the reliability and accuracy of different ML classifiers on visual screening tasks when different forms of elicited inputs are used as features.
- Evaluate the performance of the classifiers with these additional features on both balanced and imbalanced datasets—i.e., sets of images with equal and unequal proportions, respectively, of positive to negative images—of varying difficulty.
- Introduce supplementary crowdsourcing-based methods to prioritize other performance metrics of interest, namely reduced false-negative and false-positive rates.
- Analyze the performance of the crowdsourcing-based ML classifiers when outputs of an automated classifier trained on large annotated datasets are used as an additional feature.

To pursue these objectives, we design a number of experiments that elicit a diversity of inputs on each classification task: binary classification (1= positive or 0= negative); the (*x, y*)-coordinate of the target object's location; level of confidence in the binary response (on a scale from 0 to 100%); guess of what the majority of participants' binary classification is on the same task; and level of the perceived difficulty of the binary classification task (on a discrete scale). To harness the benefits of both collective human intelligence and machine intelligence, we use the elicited inputs as features for ML algorithms. The results indicate that integrating diverse forms of input elicitation, including self-reported confidence values, can improve the accuracy and efficiency of crowdsourced computation. As an additional contribution, we develop an automated image classification method based on the ResNet-50 neural network architecture (He et al., 2015) by training it on multiple datasets of sizes ranging from 10 k to 90 k image samples. The outputs of this automated classifier are used as additional features within the crowdsourcing-based ML algorithms. These additional results demonstrate that this hybrid image classification approach can provide more accurate predictions, especially for relatively larger datasets, than what is possible by either of the two stand-alone approaches.

Before proceeding, it is pertinent to mention that an earlier, shorter version of this work and a subset of its results appeared in Yasmin et al. (2021) and were presented at the 9th AAAI Conference on Human Computation and Crowdsourcing. That earlier conference paper considered only a subset of the crowdsourcing-based ML algorithms featured herein and that smaller selection was implemented only on balanced datasets. This present work also introduces a hybrid image classification approach, and it incorporates additional descriptions, crowdsourcing experiments, and analyses.

## 2. Literature Review

In recent years, crowdsourcing has been widely applied to complete a variety of image labeling/classification tasks, from those requiring simple visual identification abilities to those that rely on domain expertise. Many studies have leveraged crowdsourcing to annotate large-scale datasets, often requiring subjective analysis such as conceptualized images (Nowak and Rüger, 2010), scene-centric images (Zhou et al., 2014), and general-purpose images from publicly available sources (Deng et al., 2009; Everingham et al., 2010). Crowdsourcing techniques have also been successfully tailored to many other complex visual labeling/classification contexts that require profound domain knowledge, including identifying fish and plants (He et al., 2013; Oosterman et al., 2014), endangered species through camera trap images (Swanson et al., 2015), locations of targets (Salek et al., 2013), land covers (Foody et al., 2018), and sidewalk accessibility (Hara et al., 2012). Due to its low cost and rapid processing capabilities, another prominent use of crowdsourcing is classification of CT images in medical applications. Such tasks have included identifying malaria-infected red blood cells (Mavandadi et al., 2012), detecting clinical features of glaucomatous optic neuropathy (Mitry et al., 2016), categorizing dermatological features (Cheplygina and Pluim, 2018), labeling protein expression (Irshad et al., 2017), and various other tasks (Nguyen et al., 2012; Mitry et al., 2013).

Despite its effectiveness at processing high work volumes, numerous technical challenges need to be addressed to maximize the benefits of the crowdsourcing paradigm. One such technical challenge involves deploying effective mechanisms for judgment/estimation aggregation, that is, the combining or fusing of multiple sources of potentially conflicting information into a single representative judgment. Since the quality of the predictions is highly dependent on the method employed to consolidate the crowdsourced inputs (Mao et al., 2013), a vast number of works have focused on developing effective algorithms to tackle this task. Computational social choice is a field dedicated to the rigorous analysis and design of such data aggregation mechanisms (Brandt et al., 2016). Researchers in this field have studied the properties of various voting rules, which have been applied extensively to develop better classification algorithms. The most commonly used method across various types of tasks is Majority Voting (MV) (Hastie and Kameda, 2005). MV attains high accuracy on simple idealized tasks, but its performance tends to degrade on those that require more expertise. One related shortcoming is that MV usually elicits and utilizes only one input from each participant—typically a binary response in crowdsourcing. Relying on a single form of input elicitation may decrease the quality of the collective judgment due to cognitive biases such as anchoring, bandwagon effect, decoy effect, etc. (Eickhoff, 2018). Studies have also found that the choice of input modality, for example, using rankings or ratings to specify a subjective response, can play a significant role in the accuracy of group decisions (Escobedo et al., 2022) and predictions (Rankin and Grube, 1980). These difficulties in data collection and aggregation mechanisms become even more prominent when the task at hand is complex (e.g., see Yoo et al., 2020). Researchers have suggested many potential ways of mitigating these limitations. One promising direction is the collection of richer data, i.e., using multiple forms of input elicitation. As a parallel line of inquiry, previous works suggest that specialized aggregation methods for integrating this data should be considered for making good use of these different pieces of information (Kemmer et al., 2020).

A logical enhancement of MV for the harder tasks is to elicit the participant's level of confidence (as a proxy of expertise) and to integrate these inputs within the aggregation mechanism. In the context of group decision-making, Grofman et al. (1983) suggested weighing each individual's inputs based on self-reported confidence of their respective responses, in accordance with the belief that individuals can estimate reliably the accuracy of their own judgments (Griffin and Tversky, 1992). More recently, Hamada et al. (2020) designed a wisdom of the crowds study that asked a set of participants to rank and rate 15 items they would need for survival and used weighted confidence values to aggregate their inputs. The results were sensitive to the size of the group (i.e., number of participants); when the group was small (fewer than 10 participants), the confidence values reportedly had little impact on the results. In a more realistic application, Saha Roy et al. (2021) used binary classification and stated confidence in these inputs to locate target objects in natural scene images. Their study showed that using the weighted average of confidence values improved collective judgment. It is important to remark that these and the vast majority of related studies incorporate the self-reported confidence inputs at face value. The Slating algorithm developed by Koriat (2012) represents a different approach that determines the response according to the most confident participant. For additional uses of confidence values to make decisions, we refer the reader to Mannes et al. (2014) and Litvinova et al. (2020).

Although subjective confidence values can be a valid predictor of accuracy in some cases (Matoulkova, 2017; Görzen et al., 2019), in many others they may degrade performance owing to cognitive biases that prevent a realistic assessment of one's abilities (Saab et al., 2019). Another natural approach is to weigh responses based on some form of worker reliability. Khattak and Salleb-Aouissi (2011) used trapping questions with expert-annotated labels to estimate the expertise level of workers. For domain-specific tasks where the majority can be systematically biased, Prelec et al. (2017) introduced the Surprisingly Popular Voting method, which elicits two responses from participants: their own answer and what they think the majority of other participants' answer is. It then selects the answer that is “more popular than people predict.” Other aggregation approaches include reference-based scoring models (Xu and Bailey, 2012) and probabilistic inference-based iterative models (Ipeirotis et al., 2010; Karger et al., 2011).

In addition to crowdsourcing-based methods, automated image classification has become popular due to the breakthrough performances achieved by deep neural networks. Krizhevsky et al. (2012) used a convolutional neural network called AlexNet on a large dataset for the first time and achieved significant performance in image classification tasks compared to other contemporary methods. Since then, hundreds of studies have further improved classification capabilities, and a few have shown human-level performance when trained on large, noise-free datasets (Assiri, 2020; Dai et al., 2021). However, as the size and/or quality of training datasets decreases, the performance of these networks quickly degrades (Dodge and Karam, 2017; Geirhos et al., 2017).

A two-way relationship between AI and crowdsourcing can help compensate for some of the disadvantages associated with the two separate decision-making approaches. Human-elicited inputs interact with machine learning for a variety of reasons, but most are in service of the latter. A wider variety of ML models use human judgment to improve the accuracy and diversity in training data sets. For example, Chang et al. (2017) uses crowdsourcing to label images of cats and dogs since, unlike machines, humans can recognize these animals in many different contexts such as cartoons and advertisements. Human-elicited inputs are given more importance in specialized fields like law and medicine. For example, a study conducted by Gennatas et al. (2020) uses clinicians' inputs to improve ML training datasets and as a feedback mechanism using what is aptly termed “Expert-augmented machine learning.” In a similarly promising direction, Hekler et al. (2019) uses a combination of responses from a user study and a convolutional neural network to classify images with skin cancer; the overall accuracy of their hybrid system was higher than both components in isolation.

Unlike human-AI interaction, human-AI collaboration is an emerging focus that can lead to the formulation of more efficient and inclusive solutions. Mora et al. (2020) designed an augmented reality shopping assistant that guides human clothing choices based on social media presence, historical purchase history, etc. As part of this focus, human-in-the-loop applications seek a more balanced integration of the abilities of humans and machines by sequentially alternating a feedback loop between them. For example, Koh et al. (2017) conducted a study where a field operator wearing smart glasses uses an artificial intelligence agent for remote assistance for hardware assembly tasks. Yet, few studies seek to combine human judgments and ML outputs to form a collective decision. Developing such equitable human-AI collaboration methods could be particularly beneficial in situations where the transparency, interpretability, and overall reliability of AI-aided decisions are of paramount concern.

## 3. Crowdsourcing-Based ML Classification

This section introduces different forms of input elicitations and describes how they can be utilized within a crowdsourcing-based ML classifier. Consider the image label aggregation problem where a set of images *I* are to be labeled by a set of participants *P*; without loss of generality, assume each image and participant has a unique identifier, that is, *I* = {*i*_1_, *i*_2_, …, *i*_*n*_} and *P* = {*p*_1_, *p*_2_, …, *p*_*m*_}, where *n* and *m* represent the total number of images and participants, respectively. For each image *i*_*k*_ ∈ *I*, the objective is to infer the binary ground truth label *y*_*k*_ ∈ {0, 1}, where *y*_*k*_ = 1 if the specified target object is present in the image (i.e., positive image) and *y*_*k*_ = 0 otherwise (i.e., negative image). Since in these experiments each worker may label only a subset of the images, let *P*(*i*_*k*_) ⊆ *P* be the set of participants who complete the labeling task of image *i*_*k*_ ∈ *I*. In contrast to most crowdsourced labeling tasks where only a single label estimate is elicited per classification task, in the featured experiments each participant is asked to provide multiple inputs from the following five options. The first input is their binary response $l_{k}^{j}\in \left\{0,1\right\}$ (i.e., classification label) indicating the presence/absence of the target object in image *i*_*k*_. The second input is a coordinate-pair $\left(u_{k}^{j},v_{k}^{j}\right)$ indicating the location of the target object (elicited only when $l_{k}^{j}=1$). The third input is a numeric value $c_{k}^{j}\in \left[0,100\right]$ indicating the degree of confidence in the binary response $l_{k}^{j}$. The fourth input is another binary choice $g_{k}^{j}\in \left\{0,1\right\}$ indicating what *p*_*j*_ estimates the binary response assigned by the majority of participants to *i*_*k*_ is; this input is referred to in this study as the Guess of Majority Elicitation (GME). The fifth input is a discrete rating $d_{j}^{k}\in \left\{1,2,3,4\right\}$, whose values are mapped from four linguistic responses—1: “not at all difficult,” 2: “somewhat difficult,” 3: “very difficult,” and 4: “extremely difficult”—indicating, in increasing order, the perceived difficulty of task *i*_*k*_.

Before proceeding, it is worth motivating the use of participant confidence values in the proposed methods. Previous research has found that participants can accurately assess their individual confidence in their independently formed decisions (e.g., see Meyen et al., 2021). However, a pertinent concern regarding these confidence values is that, even if some participants are accurate in judging their performance at certain times, humans are generally prone to metacognitive biases, i.e., overconfidence or underconfidence in their actual abilities (Oyama et al., 2013). Hence, self-reported confidence should not be taken at face value, and specific confidence values should not be assumed to convey the same meaning across different individuals. In an attempt to mitigate such biases, the confidence values, ${\left\{c_{k}^{j}\right\}}_{k=1}^{n}$ provided by participant *p*_*j*_ ∈ *P* are rescaled linearly between 0 and 100, with the lowest confidence value expressed by *p*_*j*_ being mapped to 0 and the greatest to 100. Letting *I*^*j*^ ⊆ *I* be the set of images for which *p*_*j*_ provides a label, the confidence of participant *p*_*j*_ at classifying image *i*_*k*_ is rescaled as


$$c_{k}^{j*}=\frac{c_{k}^{j}-\underset{i_{q}\in I^{j}}{\min}c_{q}^{j}}{\underset{i_{q}\in I^{j}}{\max}c_{q}^{j}-\underset{i_{q}\in I^{j}}{\min}c_{q}^{j}}\times 100.$$

The remainder of this section describes how the collected input elicitations are used as features in ML classifiers to generate predictions.

### 3.1. Features for Crowdsourcing-Based ML Methods

A total of seven features were extracted from the five inputs elicitations discussed in the beginning of this section for use with the ML classifiers; these features are described in the ensuing paragraphs.

- **Binary Choice Elicitation**: For each image *i*_*k*_ ∈ *I*, the binary choice elicitation values are divided into two sets: one containing the participants with response $l_{k}^{j}=1$ and the other containing participants with response $l_{k}^{j}=0$. The number of participants in each set can be used as an input feature within a ML classifier. However, since the number of participants can vary from image to image in practical settings, it is more prudent to use the relative size of the sets. Note that these relative sizes are complements of each other, that is, the fraction of participants who chose $l_{k}^{j}=1$ as their binary choice label can be determined by subtracting from 1.0 the fraction of participants who chose $l_{k}^{j}=0$. Therefore, to remove redundancy and co-linearity within the features, only one of these values is used as an input and is given as
$$x_{k}^{1}=\frac{\underset{p_{j}\in P\left(i_{k}\right)}{\sum }1\left(l_{k}^{j}=1\right)}{\left|P\left(i_{k}\right)\right|},$$
  where $x_{k}^{1}$ is the fraction of participants who specify that the target object is present in image *i*_*k*_.
- **Spatial Elicitation**: A clustering-based approach is implemented to identify participants whose location coordinates $\left(u_{k}^{j},v_{k}^{j}\right)$—elicited only when they specify that the target object is present—are close to each other. For each image *i*_*k*_ ∈ *I*, participants with binary choice label $l_{k}^{j}=1$ are divided into multiple clusters using the Density Based Spatial Clustering of Applications with Noise (DBSCAN) algorithm (Ester et al., 1996). The reasons for choosing this algorithm are twofold. First, DBSCAN is able to identify groups of points that are close to each other but form arbitrary shapes; since the target images have varying shapes and sizes, this is what one would expect to see in a single image if all collected data points were overlaid onto it. Second, this clustering algorithm can easily mark as outliers/noise the points that are in low density areas, i.e., coordinate points that have significant distance from each other. After clustering, the fraction of participants belonging to the largest cluster is used as an input feature within the ML classifiers. For image *i*_*k*_, this input feature can be expressed as
$$x_{k}^{\text{SE}}=\frac{\underset{r\in R_{k}}{\max}n_{r}}{\underset{p_{j}\in P\left(i_{k}\right)}{\sum }1\left(l_{k}^{j}=1\right)},$$
  where *n*_*r*_ is the number of participants in cluster *r* and *R*_*k*_ is the set of clusters identified by DBSCAN for image *i*_*k*_.
- **Confidence Elicitation**: Although previous works have explored using confidence scores to improve annotation quality of crowdsourced data (Ipeirotis et al., 2010), very few have incorporated this input within a machine learning model. The confidence values are divided into two sets based on $l_{k}^{j}$, and the respective averages are used as additional features for the ML classifier. For image *i*_*k*_ ∈ *I*, these two input features can be expressed as
$$x_{k}^{\text{conf},1}=\frac{\underset{p_{j}\in P\left(i_{k}\right)}{\sum }c_{k}^{j*}1\left(l_{k}^{j}=1\right)}{\underset{p_{j}\in P\left(i_{k}\right)}{\sum }1\left(l_{k}^{j}=1\right)};\text{and}$$
$$x_{k}^{\text{conf},0}=\frac{\underset{p_{j}\in P\left(i_{k}\right)}{\sum }c_{k}^{j*}1\left(l_{k}^{j}=0\right)}{\underset{p_{j}\in P\left(i_{k}\right)}{\sum }1\left(l_{k}^{j}=0\right)}.$$
  Here, the confidence values are rescaled linearly between 0 and 100 before incorporating them as the features.
- **Guess of Majority Elicitation**: Similar to BCE, GME is converted into a single feature based on the number of participants whose $g_{k}^{j}$ response value is 1 and is written as
$$x_{k}^{\text{GME,}1}=\frac{\underset{p_{j}\in P\left(i_{k}\right)}{\sum }1\left(g_{k}^{j}=1\right)}{\left|P\left(i_{k}\right)\right|}.$$
- **Perceived Difficulty Elicitation :** Previous research has shown that a task's perceived difficulty level can be used to some extent to improve the quality of annotation. In most cases, the difficulty level is set based on inputs from experts, that is, participants with specialized knowledge with respect to the task at hand (Khattak and Salleb-Aouissi, 2011), or it is estimated from the classification labels collected from participants (Karger et al., 2011). Unlike these works, the featured experiments gather the perceived difficulty of each task directly from each participant to evaluate the reliability of this information and its potential use within ML classifiers. For each image *i*_*k*_ ∈ *I*, the difficulty elicitation values $d_{k}^{j}$ are divided into two sets: one for the participants with response $l_{k}^{j}=1$, and the other for the remaining participants with response $l_{k}^{j}=0$. The average values from each set are then used as additional features for the ML classifier; these two input features can be expressed as
$$x_{k}^{\text{PDE,}1}=\frac{\underset{p_{j}\in P\left(i_{k}\right)}{\sum }d_{k}^{j}1\left(l_{k}^{j}=1\right)}{\underset{p_{j}\in P\left(i_{k}\right)}{\sum }1\left(l_{k}^{j}=1\right)};\text{and}$$
$$x_{k}^{\text{PDE,}0}=\frac{\underset{p_{j}\in P\left(i_{k}\right)}{\sum }d_{k}^{j}1\left(l_{k}^{j}=0\right)}{\underset{p_{j}\in P\left(i_{k}\right)}{\sum }1\left(l_{k}^{j}=0\right)}.$$

## 4. Experiment Design

**Figure 1:**
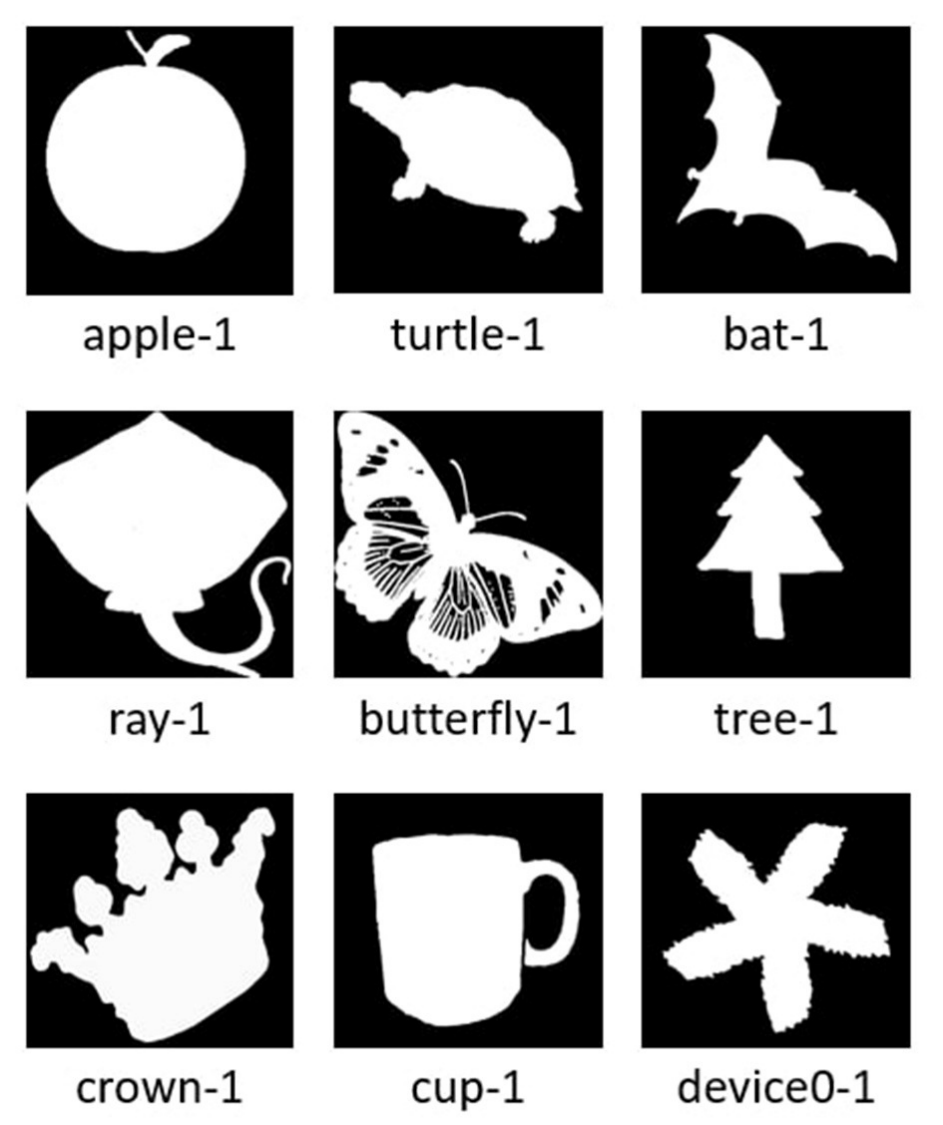
Object/shape templates from the MPEG-7 core experiment CE-shape-1 test set.

Prior to introducing the components of the experiment design, we describe the *MPEG-7 Core Experiment CE-Shape-1 Test Set* (Jeannin and Bober, 1999; Ralph, 1999), which is the source data from which the featured crowdsourcing activities are constructed. The dataset is composed of black and white images of a diverse set of shapes and objects including animals, geometric shapes, common household objects, etc. In total, the dataset consists of 1, 200 objects/shapes (referred to here as *templates*) divided into 60 object/shape classes, with each class containing 20 members. Figure 1 provides representative templates from some of these classes.

The images used in the crowdsourcing experiment are constructed by instantiating and placing multiple MPEG-7 Core Experiment CE-Shape-1 Test Set templates onto a single image frame. The instantiation of the image template is specified with six adjustable parameters: density, scale, color, transparency, rotation, and target object. See Supplementary Material for a detailed description of these parameters.

### 4.1. Description of Activities

**Figure 2:**
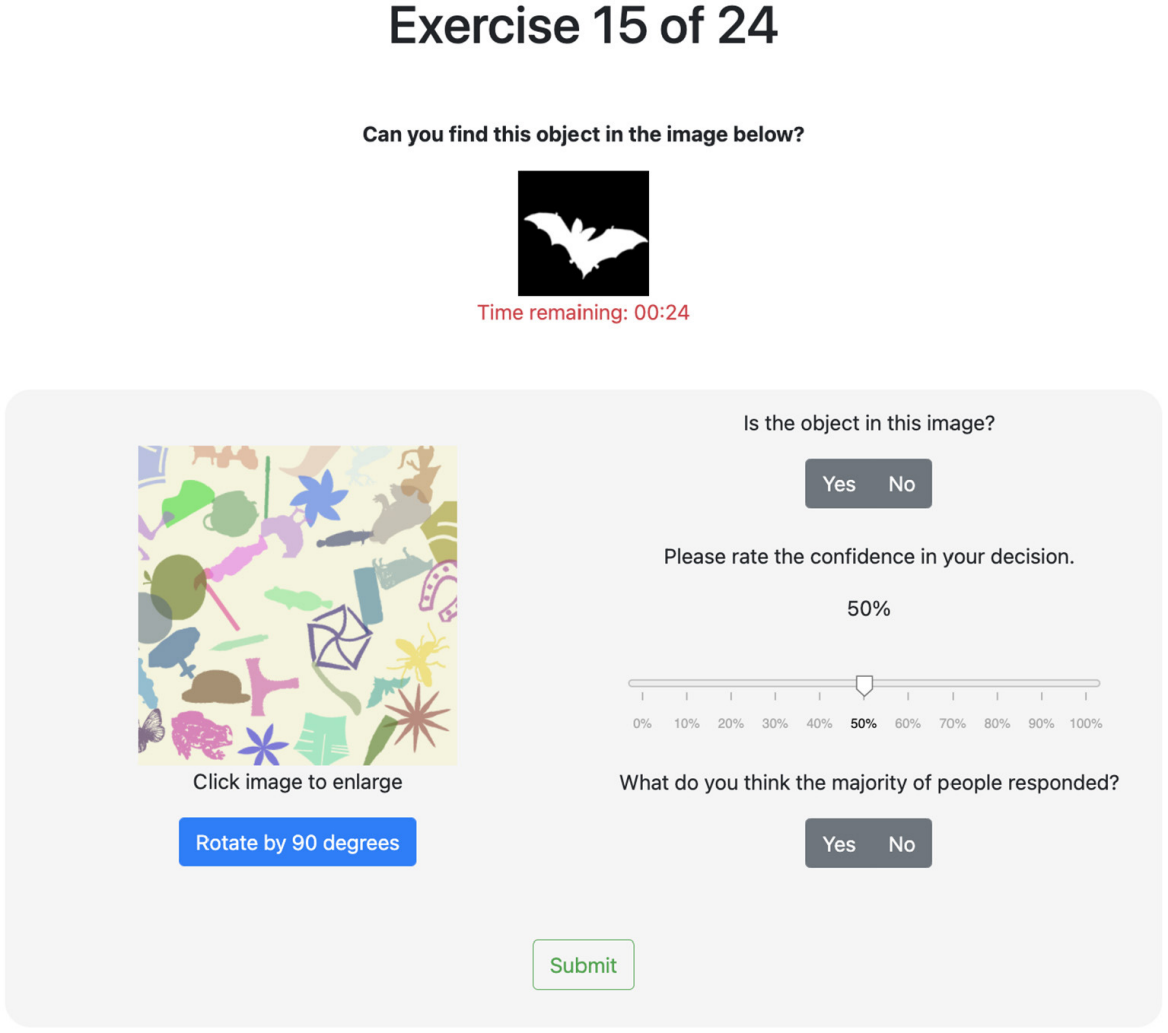
Image classification task UI for balanced dataset—image contains bat (lower right).

**Figure 3:**
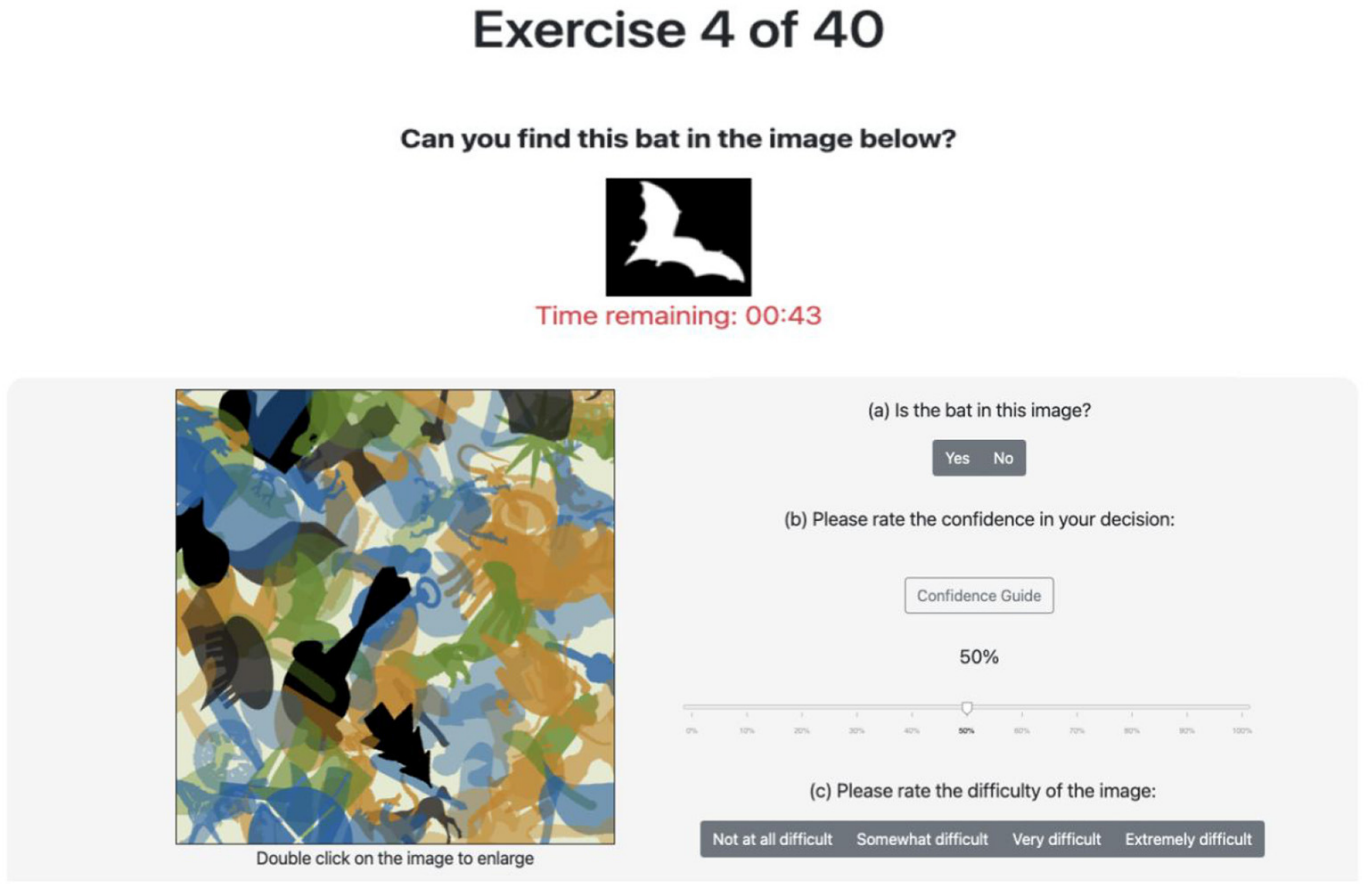
Image classification task UI for imbalanced dataset—image contains bat (center left).

For the crowdsourcing activities, we designed two studies, each of which elicits multiple forms of input from participants to complete a number of image classification tasks. A user interface was designed and implemented to perform the two studies, which differ based on the subsets of input elicitations tested and the class balance ratios of the image datasets (more details are provided later in this subsection). The interfaces were developed in HTML and Javascript and then deployed using Amazon Mechanical Turk (MTurk). Participants were first briefed about the nature of the study and shown a short walk-through video explaining the interface. Afterwards, participants proceeded to the image classification tasks, which were shown in a randomized order. After completing an experiment, participants were disallowed to participate in further experiments. Figures 2, 3 provide examples of the user interfaces, both of which instituted a 60 s time limit to view each image before it was hidden. If the participant completed the input elicitations before the time limit, they were allowed to proceed to the next image; on the other hand, if the time limit was reached, the image was hidden from view but participants could take as much time as they needed to finish providing their inputs. The time limit was imposed to ensure the scalable implementation of a high number of tasks. In particular, the goal is to develop activities that can capture enough quality inputs from participants while mitigating potential cognitive fatigue. In preliminary experiments, we found that participants rarely exceeded 45 s. In the featured studies (to be described in the next two paragraphs), the full 60 s were utilized in only 7% of the tasks, with an average time of around 27 s. The number of tasks given to the participants varied by experiment and ranged from 16 to 40 images (see Table 1 for details). We deemed this number of tasks to be reasonable and not cognitively burdensome to participants based on findings of prior studies with shared characteristics. For instance, Zhou et al. (2018) performed a visual identification crowdsourcing study where participants were assigned up to 80 tasks, each of which took a median time of 29.4 s to complete. The authors found that accuracy decreased negligibly for this workload (i.e., twice as large as in the featured studies).

**Table 1:** Summary of experiment image parameters.

**Exp**.		**Images**	**Density**	**Scale**	**Color**	**Transparency**	**Target**
SetA	#1	16	{100, 120, 140, 160}	{*T*(0.2 ± 0.12), .., *T*(0.65 ± 0.12)}	Discrete: {4}	*U*(100, 200)	Bat
	#2						Butterfly
	#3						Apple
	#4						Stingray
SetB	#5	24	{80}	{*T*(0.2 ± 0.05), *T*(0.3 ± 0.05)}	Discrete: {1,…,6}	*U*(140, 170)	Bat
	#6		{80,100,120}	{*T*(0.2 ± 0.05), .., *T*(0.4 ± 0.05)}	*U*(10, 255) for R,G,& B		Turtle
	#7		{100, 150}	{*T*(0.2 ± 0.05), *T*(0.3 ± 0.05)}			Various-7
SetC	#8	40	{90, 100, 115, 150}	{*T*(0.25, 0.35, 0.40)}	Discrete: {4}	*U*(150, 200)	Bat
	#9						
	#10						
SetD	#11						
	#12						
	#13						

In the first study, seven experiments were completed and grouped into two sets: Experiment Set A (four experiments) and Experiment Set B (three experiments). Each experiment used a balanced set of images, with half containing the target template (i.e., positive images); target objects were chosen so as to avoid confusion with other template classes. See Table 1 for image generation parameters, and see Supplementary Material for additional related details. The parameter ranges selected for Experiment Set A were designed to keep the difficulty of the classification tasks relatively moderate. On the other hand, a more complex set of parameters was selected for Experiment Set B to expand the range of difficulty. These differences are reflected in the individual performance achieved in these two experiment sets, measured by the respective average number of correct classifications obtained by participants. For Experiment Set A, individual performance averages ranged between 59 and 77% for each of the four experiments, whereas for Experiment Set B, they were between 54 and 82% for each of the three experiments.

In the second study, six experiments were conducted. These experiments were also grouped into two sets: Experiment Set C (three experiments) and Experiment Set D (three experiments). Each consisted of image sets with an imbalanced ratio of positive-to-negative images. Experiment Set C had a 20-80 balance, meaning that 20% of the images were positive, and 80% were negative; Experiment Set D had a 10–90 balance. The results of Experiment Sets A and B revealed that *scale* and *density* are the only factors that had a statistically significant impact on individual performance. Based on this insight, we constructed a simple linear regression model with these two parameters as the predictors and *proportion of correct participants* as the responses; the model is very significant (*p* < 0.001), and its adjusted R-squared value is 0.65. The model was used to generate image sets with an approximated difficulty level by modifying the scale and density parameters accordingly. It should be noted that the true difficulty of each image varies based on the random generation process. The model was implemented to design experiments consisting of classification tasks of reasonable difficulty—that is, neither trivial nor impossible to complete. Images of four levels of difficulty were generated for Experiment Sets C and D. At each difficulty level, the density was varied while keeping the other parameters consistent across images. This resulted in images that appear similar, but with different amounts of “clutter”. The four difficulties generated were categorized as “very difficult,” “difficult,” “average,” and “easy.” See Supplementary Material for details and sample images of each difficulty. Experiment Sets C and D use an even split of each difficulty (i.e., 25% of generated images from each level). For the three respective experiments, individual average accuracy values ranged between 65 and 73% for Set C and between 58 and 72% for Set D.

Figures 2, 3 show the user interface presented to participants in the first and second study, respectively. For each classification task (i.e., image) in the first study, participants were asked to provide a binary response indicating whether or not a target object is present. If they responded affirmatively, they were then prompted to locate the target object by clicking on it. Then, participants were asked to rate their confidence in their binary response on a scale from 0 to 100%. Finally, participants were asked to guess the binary response of the majority of participants. The second study asked participants similar questions as the first study. For each classification task, participants were also asked to provide a binary responses indicating whether or not a target object is present and their level of confidence in this response. If they responded affirmatively, however, they were then prompted to locate the target object by drawing a bounding box around it; the centroid of the bounding box was used as the (*x, y*)-coordinate gathered from this elicitation. In replacement to the last question of the first study, participants were asked to rate the difficulty of the specific image being classified based on a discrete scale. The rating choices provided were “not difficult at all,” “somewhat difficult,” “very difficult,” and “extremely difficult.” These labels were mapped to 1, 2, 3, and 4, respectively, for use in the aggregation algorithms.

### 4.2. Participant Demographics and Filtering of Insincere Participants

A total of 356 participants were recruited and compensated for their participation using Amazon MTurk. Participants in Experiment Set A were paid $1.25, those in Experiment Set B were paid $2.00, and those in Experiment Sets C and D were paid $3.75. The differences in compensation can be attributed to the number of questions and the difficulty of image classification tasks of the respective experiment sets. Participants were made aware of the compensation amount before beginning the study. Payment was based only on completion and not on performance. Before proceeding, it is necessary to delve further into the quality of the participants recruited *via* the MTurk platform, and the quality of data they provide. Because of the endemic presence in most crowdsourcing platforms of annotators who do not demonstrate an earnest effort (Christoforou et al., 2021), some criteria should be defined to detect such insincere participants and filter out low-quality inputs. This work defined two criteria for characterizing (and filtering out) an annotator as insincere:

- **Criterion 1:** The participant answered over 75% of the questions in no more than 10 s per question.
- **Criterion 2:** The participant's binary responses were exclusively 0 or exclusively 1 over the entire question set.

Criteria 1 was imposed based on the following reasoning. In general, classification of negative images takes longer than classification of positive images. Even if it is assumed that participants can spot the positive images immediately (i.e., within 10 s), it should take more than 10 s to reply to the negative images that are of moderate to high difficulty. Because each Experiment Set in this study contained at least 50% negative images (Experiment Sets C and D contain a higher percentage) and only a small minority were of low difficulty, a conservative estimate that participants should take longer than 10 s to answer at least 25% of the images was set (i.e., to be more lenient toward the participants). Further analysis of the behavior of the participants in relation to the task completion times supporting this observation has been added to Supplementary Material.

From the initial 356 participants, 50 participants were removed from the four experiment sets using the above criteria. Among them, 15 fell under criterion 1 and the rest under criterion 2. As expected, filtering out these data provided less noisy inputs to the crowdsourcing-based aggregation methods. From the remaining 306 participants, 276 completed the demographics survey. Their reported ages ranged from 21 to 71 years old, with a mean and median of 36 and 33, respectively. 156 participants reported their gender as male, 120 as female, and 0 as other. In terms of reported education level, 23 participants finished a high school/GED, 17 some college, 16 a 2-year degree, 148 a 4-year degree, 70 a master's degree, 1 a professional degree, and 2 a doctoral degree.

### 4.3. Distribution of Crowdsourced Data

Before proceeding to the computational results, it is pertinent to analyze the data collected from the crowdsourcing experiments. First, let us analyze the relationship between the perceived difficulty levels reported by the participants (i.e., input feature PDE) and the difficulty levels utilized in the proposed image generation algorithm (see Section 4.1 for details). The average difficulty values reported by participants for images categorized by the algorithm as “very difficult,” “difficult,” “average,” and “easy” were 2.89, 2.73, 2.62, and 2.03, respectively. This evinces a clear correlation, with the “very difficult” images having the highest average perceived difficulty values and the rest reflecting a decreasing order of difficulty, which supports the ability of the image generation method used in this study to control the classification task difficulty, according to the four above-mentioned categories.

**Figure 4:**
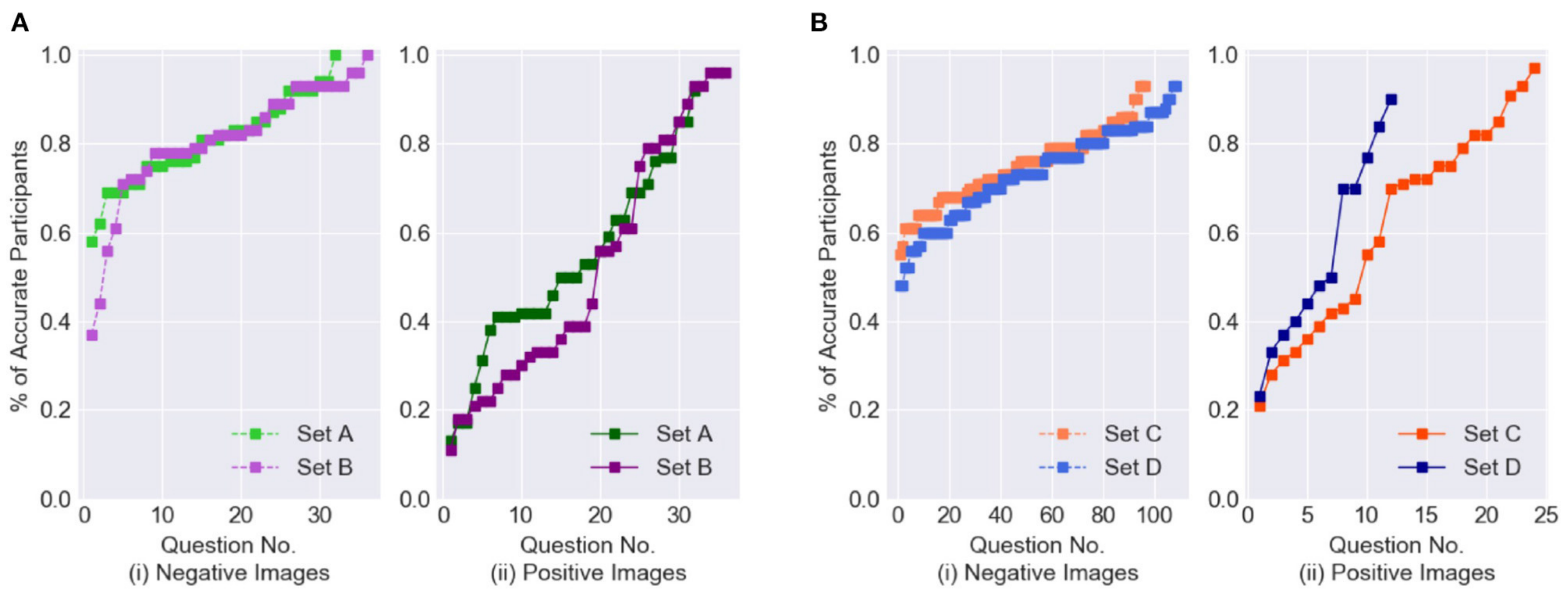
Distribution of Binary Classification results from crowdsoured data. **(A)** Balanced dataset. **(B)** Imbalanced dataset.

Next, let us analyze the correctness of the binary response values collected from the participants. Figure 4 shows the percentage of participants who answered each question accurately; question numbers have been reordered for each of the four datasets by increasing participant accuracy. The positive and negative images for the balanced and imbalanced datasets are presented in separate graphs. The plots show that, for the balanced datasets (Experiments Sets A and B), the accuracy on the positive images is significantly lower than on the negative images. Moreover, in Experiment Set B, nearly half of the positive images have accuracy values below 0.4, whereas in Experiment Set A most images have values above 0.4. This is a good indication of the higher difficulty level of Experiment Set B. For the imbalanced datasets, in both Experiments Sets C and D, nearly all negative images have accuracy values above 0.4. In Experiment Set C, there is an almost even distribution of the positive images above and below 0.6, whereas in Experiment Set D nearly 60% of the positive images have accuracy values below 0.5, indicating that Experiment Set D was comparatively more difficult.

## 5. Computational Results

This section compares the performance of the voting and crowdsourcing-based ML methods presented in Section 3 on both balanced and imbalanced datasets. As a baseline of comparison for the proposed crowdsourcing-based ML methods, three traditional voting methods are used: Majority Voting (MV), Confidence Weighted Majority Voting (CWMV), and Surprisingly Popular Voting (SPV). The details of these methods can be found in Supplementary Material. For the ML methods, four binary classification approaches were selected: K-Nearest Neighbor (KNN), Logistic Regression (LR), Random Forest Classifier (RF), and Linear Support Vector Machines (SVM-Linear). These were selected as reasonable representatives of commonly available methods. The ML classifiers were trained and evaluated using built-in functions of the Python *scikit-learn* library (Pedregosa et al., 2011). The hyper-parameters were optimized on a linear grid search with a nested 5-fold cross-validation strategy. However, due to the small size of the datasets, a Leave-One-Out (LOO) cross-validation strategy was used to train and evaluate the classifiers.

In the DBSCAN clustering approach used for extracting the Spatial Elicitation (SE), the maximum distance between two data points in the cluster (ϵ) and the minimum data points required to form a cluster (*MinPts*) was set to 50 and 3, respectively. The former was set based on the size of the target objects used relative to the size of the image frame (1, 080 × 1, 080); the latter was set to ensure a sufficiently low probability of forming a cluster with random inputs. To obtain a rough estimate of this probability, consider the case where three participants with binary response $l_{k}^{j}=1$ randomly select their location coordinates on an image with area *A*. The probability of two points having a maximum distance of *r* (i.e., falling within a circle with radius *r*) is π*r*^2^/*A* and, therefore, the probability of the three points being identified as a cluster by DBSCAN is 2(π*r*^2^/*A*)2. Setting *r* = ϵ = 50 and *A* = 1, 080 × 1, 080 for our experiment, this probability value becomes 0.01, which is sufficiently small and justifies the use of the selected parameters.

### 5.1. Performance of Aggregation Methods on Balanced Datasets

This section compares the performance of the voting and ML methods on balanced datasets (Experiment Sets A and B). The initial study elicits four out of the five inputs listed in Section 3.1: BCE, GME, CE, and SE. The results are summarized in Tables 2 and 3.

**Table 2:** Performance analysis of voting methods for balanced dataset.

	**MV**		**CWMV**		**SPV**	
	**Acc**.	**FNR**	**Acc**.	**FNR**	**Acc**.	**FNR**
Experiment Set A	0.73	0.53	0.81	0.34	0.45	0.94
Experiment Set B	0.71	0.53	0.74	0.47	0.53	0.92

**Table 3:** Performance analysis of crowdsourcing based ML methods for balanced dataset.

**Input**	**KNN**			**LR**			**RF**			**SVM-Linear**		
**Elicitations**	**Acc**.	**FNR**	**AUC**	**Acc**.	**FNR**	**AUC**	**Acc**.	**FNR**	**AUC**	**Acc**.	**FNR**	**AUC**
**Experiment Set A**												
BCE	0.83	**0.16**	0.87	**0.89**	**0.16**	**0.95**	0.83	**0.19**	0.86	**0.89**	**0.16**	0.90
BCE-CE	**0.86**	0.22	**0.89**	0.86	0.19	0.89	**0.84**	0.22	**0.93**	0.86	0.19	0.91
BCE-SE	0.84	0.22	0.85	0.88	**0.16**	0.91	0.83	0.22	0.87	0.86	0.19	0.92
BCE-GME	**0.86**	0.19	0.87	0.86	**0.16**	0.91	0.83	**0.19**	0.83	0.88	**0.16**	0.91
BCE-CE-SE	0.81	0.31	0.86	0.88	0.19	0.88	**0.84**	0.22	0.91	**0.89**	**0.16**	0.91
BCE-CE-GME	0.8	0.25	0.82	0.84	0.19	0.90	0.83	0.22	0.89	0.84	0.19	0.90
BCE-CE-SE-GME	**0.86**	0.25	0.82	0.83	0.19	0.90	0.83	0.22	0.89	0.86	0.19	0.89
**Experiment Set B**												
BCE	0.75	0.28	0.79	0.81	0.28	0.74	0.75	0.31	0.76	0.74	0.42	0.85
BCE-CE	0.78	0.28	**0.85**	0.81	0.25	0.88	0.75	**0.22**	**0.82**	**0.79**	**0.25**	0.85
BCE-SE	**0.79**	**0.19**	0.81	0.68	0.42	0.55	0.74	0.31	0.78	0.74	0.44	0.80
BCE-GME	0.75	0.31	0.78	0.76	0.31	**0.89**	0.68	0.33	0.74	0.72	0.42	**0.88**
BCE-CE-SE	0.76	0.22	0.79	**0.82**	**0.22**	**0.89**	0.74	0.25	0.80	0.72	0.47	0.85
BCE-CE-GME	0.76	0.31	0.81	0.78	0.28	0.80	**0.76**	**0.22**	0.79	0.78	0.31	0.86
BCE-CE-SE-GME	0.72	0.36	0.82	0.78	0.31	0.87	0.72	0.31	0.79	0.74	0.47	0.83

*Bold values denote best performance among the different input elicitation combinations for each Crowdsourcing-based ML method*.

The performance of the ML methods is quantified *via* three performance metrics: accuracy (Acc.), false-negative rate (FNR), and area under the ROC curve (AUC). For the voting methods, only the first two of these metrics are reported. For each of the ML classifiers, the best accuracy, FNR, and AUC values among the different input elicitation combinations are marked in bold. Before proceeding, it is worthwhile to mention two additional points regarding the values presented in the table. First, each row in Table 3 represents a different combination of inputs used as features for the ML classifiers. For example, BCE-CE indicates that both binary and confidence elicitation inputs (i.e., $x_{k}^{1},x_{k}^{\text{conf},1}$ and $x_{k}^{\text{conf},0}$) were used as features for the ML classifiers, whereas BCE-CE-SE-GME indicates that all four input elicitations (i.e., $x_{k}^{1},x_{k}^{\text{conf},1},x_{k}^{\text{conf},0},x_{k}^{\text{SE}}$, and $x_{k}^{\text{GME,}1}$) of Experiment Set A and B were used as the respective input features. Second, when calculating the accuracy and FNR values of the voting methods, images with undecided outcomes (i.e., ties) are considered as a third separate label.

Let us first discuss the performance of the aggregation models in terms of accuracy. For Experiment Sets A and B, the average accuracy value of MV was stable at around 72%. The CWMV method performed significantly better than MV, achieving an average accuracy value of around 77%. SPV was the worst performer across the board, with an average accuracy value of <50% (i.e., worse than a purely random classifier). This low performance can be largely attributed to the excessive number of tied labels generated compared to the other methods. In SPV, 18 out of the 136 instances were classified as tied (i.e., participants were undecided regarding the guess of the majority's estimate). By comparison, there were only three tied instances with MV and none with CWMV.

The results of the ML classifiers in Experiment Set A were relatively consistent in terms of both accuracy and AUC values for all seven combinations of the input elicitations. The classifiers performed particularly well, attaining accuracy values above 83% for all combinations; this can be partly explained by the fact that the images in this experiment set were generated using parameter ranges that were more consistent and less variable in difficulty. In Experiment Set B, the ML classifiers reached higher accuracy and AUC values under certain combinations of the input elicitations. For RF, LR, and KNN, a noticeable increase in AUC values (from 76 to 85%) results when using the BCE-CE combination compared to the standalone BCE input; the accuracy values in these cases either increased or stayed the same. Altogether, these results suggest that integrating CE into an ML classifier can help attain more accurate predictions when the sample size is small and the difficulty level of the images is more varied. Furthermore, they show that the ML classifiers outperformed the voting methods, with the LR classifier achieving the highest values in terms of both accuracy and AUC scores.

Another performance metric of interest is FNR, which denotes the fraction of images the methods label as 0 (i.e., negative) when their true label is 1 (i.e., positive). A high FNR may be concerning in many critical engineering and medical applications where a false-negative may be more detrimental than a false-positive since the latter can be easily verified in subsequent steps. For example, FNR has significant importance in detecting lung cancer from chest X-rays. If the model falsely classifies an X-ray as negative, the patient may not receive needed medical care in a timely fashion. Returning to Table 2, the FNRs of the three voting methods are high across the board, with SPV again having the worst performance. The high FNRs of MV and CWMV can be attributed to the fact that people tend to label the image as negative whenever they fail to find the target object and that these methods are unable to extract additional useful information from the responses.

In Experiment Set A, the accuracy values are the highest for the BCE-CE combination, whereas the FNR values are the lowest for the single BCE input. On the other hand, in Experiment Set B, although the accuracy values are the same for both input combinations, FNR values decrease for the BCE-CE combination. Moreover, for SVM, the reduction in FNR values is significant for Experiment Set B (from 42 to 25%) for the BCE-CE combination. This outcome reiterates the advantages of integrating CE into ML classifiers for more complex datasets.

### 5.2. Performance of Aggregation Methods on Imbalanced Datasets

This section compares the performance of the voting and crowdsourcing-based ML methods on imbalanced datasets (Experiment Sets C and D). Similar to the balanced datasets, a total of four input elicitations are utilized. However, for this study, the GME input is replaced by the PDE input (i.e., a rating value to assess the difficulty of the classification task), which is explained as follows. Recall from the discussion of Section 5.1 that none of the ML classifiers obtained a performance improvement when using the GME input relative to the other elicitation combinations. Moreover, the only method that utilizes the GME elicitation, SPV, was the worst-performing among the three voting methods. The inability of the GME input to provide any additional information during the classification process prompted its removal from subsequent studies. Due to this modification, only two voting methods (MV and CWMV) are explored for the imbalanced datasets.

When the dataset is balanced, accuracy by itself is a good indicator of the model's performance. However, when the dataset is imbalanced, accuracy can often be misleading as it provides an overly optimistic estimation of the classifier's performance on the majority class (“0” in this experiment). In such cases, a more accurate evaluation metric is the *F*_1_-score (Sokolova et al., 2006), defined as the harmonic mean of the precision and recall values and can be expressed as, *F*_1_-$\text{score}=2\left(\text{Precision}\times \text{Recall}\right)/\left(\text{Precision}+\text{Recall}\right)=\text{TP}/\left[\text{TP}+\frac{1}{2}\left(\text{FP}+\text{FN}\right)\right],$ where, TP, FP, and FN refers to the number of true-positives (images the methods label as 1 when their true label is 1), false-positives (images the methods label as 1 when their true label is 0), and false-negatives (images the methods label as 0 when their true label is 1), respectively. Since both Experiment Sets C and D are highly imbalanced (with an average of 15% of their images belonging to the positive class) the *F*_1_-score is reported instead of accuracy to better estimate the performance of the classifiers.

The overall results for the voting and machine learning methods are summarized in Tables 4, 5, respectively. The performance of the ML methods is quantified *via* three performance metrics: *F*_1_-score, FNR, and AUC; for the voting methods, only the first two of these metrics are reported. Let us first discuss the performance of the aggregation methods in terms of *F*_1_-score. For Experiment Sets C and D, MV and CWMV have comparable scores, with both having the same value in the first set and MV outperforming CWMV by a slight margin in the second set. Moving on to the ML methods, for Experiment Set C, the ML classifiers displayed comparable *F*_1_-scores for combinations BCE-CE, BCE-SE, BCE-CE-SE, and BCE-CE-SE-PDE. In addition, all four of these input combinations performed better than the standalone BCE input. The RF and KNN classifiers achieved the highest values with the combination BCE-CE-SE-PDE. In contrast, the LR and SVM classifiers achieved the highest values with the BCE-SE combination. Overall, the LR classifier achieved the best performance for this set with inputs BCE-SE. In Experiment Set D, the results followed a different pattern. In this case, the classifiers achieved the same or higher values when the BCE-CE combination was used compared to the BCE-SE or BCE-CE-SE combinations, indicating that the SE input does not provide any additional information for this experiment set. Because this dataset is highly skewed toward the negative class (10–90 balance), we conjecture that participants may have become demotivated to closely inspect difficult images from the positive class. Whatever the cause, smaller clusters were obtained from these images, reducing the effectiveness of the SE input in many cases. In Experiment Set D, the highest performance was achieved by the SVM classifier for the BCE-CE input. These results once again indicate that, even though the self-reported confidence values are not particularly helpful when used within the traditional voting methods context (Li and Varshney, 2017; Saab et al., 2019)—as can also be seen by the performance of the CWMV algorithm in this study—incorporating them into an ML classifier can help attain better performance, specifically higher *F*_1_-scores for highly imbalanced datasets.

**Table 4:** Performance analysis of voting methods for imbalanced dataset.

	**MV**		**CWMV**	
	***F*_1_**	**FNR**	***F*_1_**	**FNR**
Experiment Set C	0.77	0.38	0.77	0.25
Experiment Set D	0.53	0.58	0.52	0.50

**Table 5:** Performance analysis of crowdsourcing based ML methods for imbalanced datasets.

**Input** **Elicitations**	**KNN**			**LR**			**RF**			**SVM-Linear**		
	***F*_1_**	**FNR**	***AUC***	***F*_1_**	**FNR**	**AUC**	***F*_1_**	**FNR**	**AUC**	***F*_1_**	**FNR**	**AUC**
**Experiment Set C**												
BCE	0.73	0.38	0.82	0.78	0.33	0.79	0.73	0.33	0.81	0.73	0.38	0.92
BCE-CE	0.75	0.38	0.89	0.81	0.29	0.90	0.78	0.33	0.86	0.80	0.33	0.90
BCE-SE	**0.81**	**0.29**	0.83	**0.84**	**0.25**	**0.95**	0.76	**0.29**	0.87	**0.84**	**0.25**	0.86
BCE-PDE	**0.81**	**0.29**	0.83	0.76	0.33	0.94	0.68	0.38	0.81	0.77	0.38	0.9
BCE-CE-SE	0.76	0.33	**0.92**	0.81	0.29	0.92	0.77	**0.29**	**0.90**	0.81	0.29	0.88
BCE-CE-PDE	**0.81**	**0.29**	0.90	0.81	0.29	0.86	0.79	**0.29**	0.86	0.80	0.33	0.90
BCE-CE-SE-PDE	**0.81**	**0.29**	**0.92**	0.81	0.29	0.86	**0.81**	**0.29**	**0.90**	0.81	0.29	0.86
**Experiment Set D**												
BCE	0.53	**0.58**	0.59	0.55	**0.33**	**0.87**	0.36	0.58	0.64	0.61	**0.42**	0.85
BCE-CE	**0.59**	**0.58**	**0.76**	0.54	0.42	0.83	**0.63**	**0.50**	**0.79**	**0.67**	0.50	**0.87**
BCE-SE	**0.59**	**0.58**	0.62	0.46	0.50	0.84	0.36	0.58	0.65	0.63	0.50	0.8
BCE-PDE	0.50	0.67	0.67	**0.57**	**0.33**	0.85	0.47	0.67	0.78	0.56	**0.42**	0.86
BCE-CE-SE	**0.59**	**0.58**	0.72	0.52	0.42	**0.87**	0.53	0.58	0.77	**0.67**	0.50	0.84
BCE-CE-PDE	0.44	0.67	0.68	0.56	0.42	0.73	0.53	0.58	**0.79**	0.63	0.50	**0.87**
BCE-CE-SE-PDE	0.56	**0.58**	0.74	0.52	0.42	0.84	0.44	0.67	0.78	**0.67**	0.50	0.85

*Bold values denote best performance among the different input elicitation combinations for each Crowdsourcing-based ML method*.

In terms of FNR, the performance of the CWMV method was markedly better than the MV method for both Experiment Sets. The assigned labels for the positive images in Experiment Set D for the two voting methods are almost identical, with the exception of a single image which the latter labeled as a tie (i.e., undecided), contributing to the decrease in performance. Note that none of the images in Experiment Set C was labeled as a tie by either of the voting methods. Among the ML methods, LR significantly outperformed all of the other classifiers for Experiment Set D. Although in Experiment Set C the FNR for the BCE-SE combination (25%) was lower than for the BCE combination (33%), in Experiment Set D a significant increase (33–50%) can be seen between these two combinations. Overall, ML classifiers outperformed MV; however, CWMV showed comparable performance for both experiment sets. Note that a distinctive advantage of CWMV over the ML methods is that it does not require training data.

### 5.3. Changing the Threshold of Positive Classification

This section examines how voting methods can be modified to emphasize other important metrics of image classification. In particular, it seeks to prioritize reduced false-negative rates, which are relevant in various critical applications. The FNRs can be reduced by lowering the threshold at which a positive classification is returned by a classification method (i.e., changing the tipping point for returning a positive collective response). However, care must be exercised when lowering the threshold since this implicitly increases false-positive rates (FPRs), which can also be problematic.

**Figure 5:**
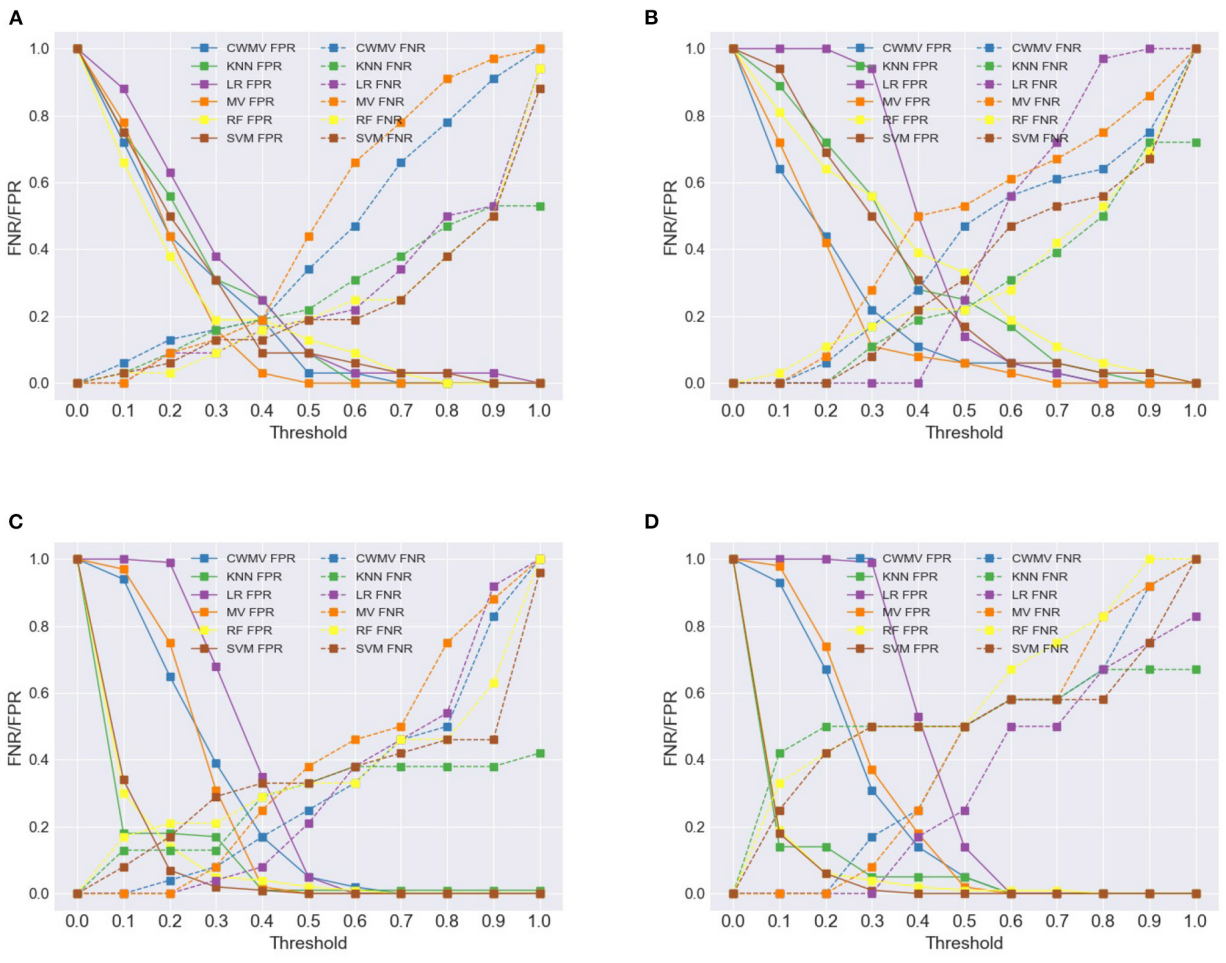
Change in FNR/FPR of different aggregation methods under varying thresholds. **(A)** Experiment Set A, **(B)** Experiment Set B, **(C)** Experiment Set C, **(D)** Experiment Set D.

By default, the threshold at which voting methods return a positive response is fixed; for example, MV requires more than 50% of positive responses to return the positive class. Figure 5 illustrates the impacts of adjusting the thresholds for the voting methods as well as for the ML methods; the figure separates FNRs from FPRs for each method. Using MV as an example, decreasing the threshold from 0.5 to 0.3 results in relatively small increases to the FPR and larger decreases to the FNR; further decreases cause a disproportionate increase to FPRs. Hence, these inflection points can help guide how the thresholds can be set for each voting method to prioritize FNR. A similar observation can be made about the FNRs of the ML methods (except for LR) for the imbalanced datasets. However, this does not hold for the ML methods for the balanced datasets—for example, reducing the threshold to 0.3 causes a significant increase in FPRs compared to the decrease in FNRs. This suggests that caution must be exercised when changing the threshold of positive classification of ML classifiers.

## 6. Enhancement of Crowdsourcing-Based ML Methods With an Automated Classifier

In order to assess the difficulty of the image classification problem presented to participants and to evaluate the potential of hybrid human-ML approaches, we developed a deep learning image classification approach that leverages large training datasets. Our classifier is based on ResNet-50, a popular variant of ResNet architecture (He et al., 2015), which has shown very good performance on multiple image classification tasks. It has been extensively used by the computer vision research community and adopted as a baseline architecture in many studies done over the last few years (Bello et al., 2021).

For training the classifier, we generated a balanced dataset of 100 k samples, with 10 k samples set aside as the validation set and the rest used as the training set. The images are representative of an even mixture of the difficulty classes used to generate Experiment Sets C and D. We trained and evaluated the performance of the network using training set sizes ranging from 10 k samples to 90 k samples, increasing the training set size by 10 k every iteration, totaling nine different training sessions. Each training session was started from the previous session's best-performing checkpoint of the network and the corresponding optimization state and continued for 35 epochs. See Supplementary Material for a complete description of the ResNet classifier used as well as a detailed analysis of its performance.

We emphasize that this work does not aim to advance the state-of-the-art results for automated image classification. Instead, the focus of the automated classification method is to explore the benefits and limitations of a hybrid method introduced herein that integrates the outputs of a well-known deep neural network into the crowdsourcing-based classification methods. In particular, the proposed method uses the output of the automated classifier as an additional feature of the featured ML methods. Table 6 summarizes the results for the small imbalanced test sets used in Experiment Sets C and D as the training set grows larger. Due to the imbalanced nature of these test sets, this table and the rest of the analysis focus on *F*_1_-score, false-negative rate (FNR), and area under the ROC curve (AUC). Before proceeding, it is worthwhile to mention two additional points regarding the values presented in the table. First, the input elicitation RC represents the probability value of positive classification obtained from the automated classifier when used as a feature. For example, BCE-RC indicates that both the binary elicitation inputs and the probability scores from the ResNet-50 were used as features for the ML classifiers. Second, the Combined Set C&D is created by merging the data from Experiment Sets C and D, thereby effectively doubling the size of the training set relative to the individual experiment sets.

**Table 6:** Performance analysis of Crowdsourcing-based ML methods with expanded inputs from ResNet-50.

**Input** **Elicitations**	**Size of** **dataset**	**ResNet50**			**KNN**			**LR**			**RF**			**SVM-Linear**		
		***F*_1_**	**FNR**	**AUC**	***F*_1_**	**FNR**	**AUC**	***F*_1_**	**FNR**	**AUC**	***F*_1_**	**FNR**	**AUC**	***F*_1_**	**FNR**	**AUC**
**Experiment Set C**																
BCE-CE-SE-PDE^*^	–	–	–	–	0.81	0.29	0.92	0.81	0.29	0.86	0.81	0.29	0.90	0.81	0.29	0.86
RC	10k	0.36	0.21	0.67	–	–	–	–	–	–	–	–	–	–	–	–
BCE-RC		–	–	–	0.73	0.38	0.85	**0.82**	0.25	**0.93**	0.70	0.38	0.89	0.75	0.38	0.92
BCE-CE-RC		–	–	–	0.70	0.42	0.89	0.77	0.25	0.88	0.74	0.33	0.86	0.80	0.33	0.91
RC	30k	0.71	0.29	0.92	–	–	–	–	–	–	–	–	–	–	–	–
BCE-RC		–	–	–	0.77	0.38	0.83	0.75	**0.25**	0.92	0.78	0.33	0.89	0.76	0.33	0.92
BCE-CE-RC		–	–	–	0.75	0.38	0.81	0.73	**0.25**	0.91	0.78	0.33	0.88	0.80	0.33	**0.93**
RC	50k	0.87	0.04	0.99	–	–	–	–	–	–	–	–	–	–	–	–
BCE-RC		–	–	–	0.80	0.25	0.95	**0.90**	0.04	0.97	0.84	0.21	0.97	**0.88**	0.13	0.98
BCE-CE-RC		–	–	–	0.82	0.25	0.92	**0.90**	0.04	0.98	0.84	0.21	0.97	**0.88**	0.13	0.97
RC	70k	0.90	0.08	0.99	–	–	–	–	–	–	–	–	–	–	–	–
BCE-RC		–	–	–	**0.91**	0.13	0.98	**0.92**	**0.04**	0.99	**0.93**	0.13	0.98	**0.94**	**0.04**	0.99
BCE-CE-RC		–	–	–	**0.91**	0.13	0.98	0.88	**0.04**	**1.00**	**0.93**	0.13	0.98	**0.94**	**0.04**	0.99
RC	90k	0.96	0.00	1.00	–	–	–	–	–	–	–	–	–	–	–	–
BCE-RC		–	–	–	**0.98**	0.04	0.98	0.94	0.04	0.96	**0.98**	0.04	0.97	**0.98**	0.04	0.99
BCE-CE-RC		–	–	–	**0.98**	0.04	0.98	0.9	0.04	0.97	**0.98**	0.04	0.97	**0.98**	0.04	0.99
**Experiment Set D**																
BCE-CE^*^	–	–	–	–	0.59	0.58	0.76	0.54	0.42	0.83	0.63	0.50	0.79	0.67	0.50	0.87
RC	10k	0.17	0.33	0.62	–	–	–	–	–	–	–	–	–	–	–	–
BCE-RC		–	–	–	0.59	0.58	0.67	0.44	**0.25**	0.87	0.44	0.67	0.73	0.11	0.42	0.78
BCE-CE-RC		–	–	–	0.56	0.58	0.69	0.43	0.33	0.84	0.63	0.50	0.78	0.63	0.50	0.86
RC	30k	0.50	0.42	0.87	–	–	–	–	–	–	–	–	–	–	–	–
BCE-RC		–	–	–	0.50	0.67	0.67	0.43	**0.33**	0.87	0.59	0.58	0.74	0.63	0.50	0.85
BCE-CE-RC		–	–	–	0.50	0.67	0.64	0.47	0.42	**0.88**	0.56	0.58	0.8	0.67	0.50	0.87
RC	50k	0.79	0.08	0.96	–	–	–	–	–	–	–	–	–	–	–	–
BCE-RC		–	–	–	0.74	0.42	0.90	0.71	0.17	0.91	0.70	0.42	0.88	**0.80**	0.17	0.96
BCE-CE-RC		–	–	–	0.70	0.42	0.91	0.69	0.17	0.90	0.74	0.42	0.86	**0.80**	0.17	0.91
RC	70k	0.83	0.17	0.98	–	–	–	–	–	–	–	–	–	–	–	–
BCE-RC		–	–	–	**0.91**	0.17	0.96	**0.88**	**0.08**	0.92	**0.91**	0.17	0.94	**0.96**	**0.08**	0.92
BCE-CE-RC		–	–	–	**0.91**	0.17	0.96	**0.88**	**0.08**	0.92	**0.91**	0.17	0.93	**0.96**	**0.08**	0.92
RC	90k	0.96	0.08	0.98	–	–	–	–	–	–	–	–	–	–	–	–
BCE-RC		–	–	–	0.96	0.08	0.96	0.92	0.08	0.94	0.91	0.17	0.94	0.96	0.08	0.95
BCE-CE-RC		–	–	–	0.96	0.08	0.96	0.92	0.08	0.95	0.91	0.17	0.94	0.96	0.08	0.92
**Combined Set C&D**																
BCE-CE^*^	–	–	–	–	0.68	0.47	0.83	0.73	0.33	0.9	0.72	0.42	0.85	0.76	0.39	0.9
RC	10k	0.27	0.25	0.65	–	–	–	–	–	–	–	–	–	–	–	–
BCE-RC		–	–	–	0.67	0.5	0.83	0.64	0.25	0.88	0.67	0.39	0.86	0.71	0.39	**0.91**
BCE-CE-RC		–	–	–	0.69	0.44	0.86	0.68	0.25	0.9	0.69	0.44	0.84	0.76	0.39	**0.91**
RC	30k	0.63	0.33	0.90	–	–	–	–	–	–	–	–	–	–	–	–
BCE-RC		–	–	–	0.71	0.42	0.87	0.66	**0.25**	**0.92**	**0.79**	**0.31**	**0.94**	0.72	0.42	**0.91**
BCE-CE-RC		–	–	–	0.72	0.42	0.84	0.64	**0.28**	**0.92**	0.75	0.39	**0.91**	0.72	0.42	**0.93**
RC	50k	0.84	0.06	0.98	–	–	–	–	–	–	–	–	–	–	–	–
BCE-RC		–	–	–	**0.87**	0.19	0.96	**0.86**	0.08	0.97	**0.91**	0.11	0.96	**0.85**	0.14	0.97
BCE-CE-RC		–	–	–	**0.86**	0.22	0.94	**0.86**	0.08	0.96	**0.86**	0.22	0.96	**0.85**	0.14	0.98
RC	70k	0.88	0.11	0.99	–	–	–	–	–	–	–	–	–	–	–	–
BCE-RC		–	–	–	**0.96**	**0.08**	0.97	**0.92**	**0.06**	0.94	**0.96**	**0.08**	0.96	**0.93**	**0.06**	0.97
BCE-CE-RC		–	–	–	**0.93**	**0.08**	0.97	**0.89**	**0.06**	0.97	**0.96**	**0.08**	0.96	**0.92**	**0.06**	0.97
RC	90k	0.96	0.03	0.99	–	–	–	–	–	–	–	–	–	–	–	–
BCE-RC		–	–	–	**0.97**	0.06	0.97	0.93	0.06	0.98	**0.97**	0.06	0.96	**0.97**	0.06	0.98
BCE-CE-RC		–	–	–	**0.97**	0.06	0.97	0.92	0.06	0.94	**0.97**	0.06	0.97	**0.97**	0.06	0.98

*Denotes the input combinations that achieved the best performance among the Crowdsourcing-based ML methods. Bold values denote cases where hybrid method outperforms both the Resnet-50 classifier and the Crowdsourcing-based ML methods.

Table 6 marks in bold those cases in which the performance of the hybrid method according to a given metric is better than both the completely automated approach (ResNet-50) and the results achieved by the crowdsourcing-based ML methods (according to the best input combination). As expected, when the ResNet-50 performance is poor, using its output as a feature hurts the overall results. Conversely, when the ResNet-50 performance is near perfect, it is difficult to improve upon its performance by adding information obtained from the crowd. However, apart from those extremes, exploiting the output of the ResNet-50 is beneficial in most cases, particularly regarding *F*_1_-score and AUC.

The proposed hybrid methods, which use the results from the automated classifier as an additional input feature for the crowdsourcing-based ML methods, exhibited a robust performance. They attained maximum *F*_1_-scores of 0.98, 0.96 0.97 and minimum FNRs of 0.04, 0.08, 0.06 for Experiment Set C, D, and Combined Set C&D, respectively, all of which represent significant improvements over what crowdsourcing-based methods achieved on a standalone basis. While these top results were associated with the automated classifier training set of 90k samples, impressive results were obtained using smaller datasets for Combined Set C&D, compared to Experiment Set C and D separately. As an example, incorporating the output of the automated classifier trained on 50k samples with the crowdsourcing-based methods for Combined Set C&D improved the *F*_1_-score significantly (see Tables 5, 6). However, the hybrid approach did not show better results for Experiment Sets C and D separately over the same training set size in some cases. This can be explained by the fact that Experiment Sets C and D have fewer data points than Combined Set C&D. This attests that, while crowdsourcing-based methods supplemented with the outputs of the automated classifier perform very well on small datasets, too few data points can negatively affect the performance of the hybrid approach.

## 7. Discussion

This section highlights key observations related to the research questions, along with the limitations of the study. The experiment results demonstrate that supplementing binary choice elicitation with other forms of inputs can generate better classifiers. When the training sets is small, incorporating binary labels along with confidence values regarding these responses within any of the four ML classifiers tested in this work generated more dependable results for datasets of varying levels of difficulty. These diverse inputs also helped improve other performance metrics such as AUC values, which measure an ML model's capability to distinguishing between labels. While voting methods had a rather poor performance with respect to FNRs, a simple parametric modification (i.e., changing the threshold value) was shown to significantly reduce these values with comparatively small increases to FPRs. When the training sets is larger, integrating the inputs from the automated classifier with the crowdsourcing-based ML methods decreased FNRs even further. Those methods achieved near-perfect FNRs thanks to a large dataset that was used to train the automated classifier. The *F*_1_-score was also improved significantly through this hybrid approach. Although smaller training sets of 50k samples slightly reduced the performance of the automated classifier, the numbers were still better than those obtained by standalone crowdsourcing-based methods. Altogether, the results demonstrate that including diverse inputs as features within an ML classifier, it is possible to obtain better classifications at a relatively low cost.

The methodology for aggregating crowd information to improve image classification outcomes presented in this paper could have wide-ranging applications. Through suitable adaptations and enhancements, it could be applied for various types of real-world screening tasks, such as inspecting luggage at travel checkpoints (e.g., airports, metro), X-ray imaging for medical diagnosis, online image labeling, AI model training using CAPTCHAs, etc. Moreover, the image classification problem featured herein is a special case of the overall participant information aggregation problem; therefore, the findings in this paper could be extended to various other classification applications that utilize the wisdom of the crowd concept.

The presented studies admittedly have some limitations. For starters, the approach used to filter “insincere participants" was relatively simple. To obtain a better quality dataset, future studies will seek to deploy more sophisticated quality control techniques for filtering out unreliable or poor quality participants, e.g., using Honeypot questions (Mortensen et al., 2017). A second limitation is that the synthetic images generated for this work have certain characteristics that may overly benefit automated classification methods but may not generalize to various real-world situations. It is possible, for example, that the images might have tiny consistent details that are not visible to human eyes due to the nature of the image generation process. In that case, the automated classification method had an unfair advantage of exploiting those details to improve performance effectively. Future studies will assess the featured methods on more realistic datasets drawn from other practical contexts.

## 8. Conclusion

Although crowdsourcing methods have been productive in image classification, they do not tap into the full potential of the wisdom of the crowd in one important respect. These methods have largely overlooked the fact that difficult tasks can be amplified to elicit and integrate multiple inputs from each participant; an easy-to-implement option, for example, is eliciting the level of confidence in one's binary response. This paper investigates how different types of information can be utilized with machine learning to enhance the capabilities of crowdsourcing-based classification. It makes four main contributions. First, it introduces a systematic synthetic image generation process that can be used to create image classification tasks of varying difficulty. Second, it demonstrates that while reported confidence in one's response does not significantly raise the performance of voting methods, this intuitive form of input can enhance the performance of machine learning methods, particularly when smaller training datasets are available. Third, it explains how aggregation methods can be adapted to prioritize other metrics of interest of image classification (e.g., reduced false-negative rates). Fourth, it demonstrates that under the right circumstances, automated classifiers can significantly improve classification performance when integrated with crowdsourcing-based methods.

The code used to generate the synthetic images can be found at https://github.com/O-ARE/2D-Image-Generation-HCOMP. In addition, the code used to train and evaluate the automated classifier can be found at https://github.com/O-ARE/2d-image-classification.

## Data Availability Statement

The raw data supporting the conclusions of this article can be made available by the authors upon request.

## Ethics Statement

The studies involving human participants were reviewed and approved by Tiffany Dunning, IRB Coordinator, Arizona State University. The patients/participants provided their written informed consent to participate in this study.

## Author Contributions

RY, AE, OF, MH, and JG contributed to the conception and design of the study. HB organized the database. RY, JG, and MH performed the computational analysis. RY wrote the first draft of the manuscript. MH, JG, and HB wrote sections of the manuscript. All authors contributed to manuscript revision, read, and approved the submitted version.

## Funding

This material was based upon work supported by the U.S. Department of Homeland Security under Grant Award Number 17STQAC00001-05-00, which all authors gratefully acknowledge. The lead PI of the project (AE) and two of the students (JG and HB) also gratefully acknowledge support from the National Science Foundation under Award Number 1850355. An earlier, shorter version of this paper and a smaller subset of the results featured herein have been published in Yasmin et al. (2021) and presented in the 9th AAAI Conference on Human Computation and Crowdsourcing.

## Author Disclaimer

The views and conclusions contained in this document are those of the authors and should not be interpreted as necessarily representing the official policies, either expressed or implied, of the U.S. Department of Homeland Security or of the National Science Foundation.

## Conflict of Interest

The authors declare that the research was conducted in the absence of any commercial or financial relationships that could be construed as a potential conflict of interest.

## Publisher's Note

All claims expressed in this article are solely those of the authors and do not necessarily represent those of their affiliated organizations, or those of the publisher, the editors and the reviewers. Any product that may be evaluated in this article, or claim that may be made by its manufacturer, is not guaranteed or endorsed by the publisher.
