# Supplementary material for: Improving Crowdsourcing-Based Image Classification Through Expanded Input Elicitation and Machine Learning
Source: Front Artif Intell. 2022 Jun 29;5:848056. doi: 10.3389/frai.2022.848056 (PMC9276979; doi:10.3389/frai.2022.848056)
Supplement: Supplementary file 1 [file Data_Sheet_1.PDF]

frontiers

Supplementary Material

1 IMAGE GENERATION PROCESS

The instantiation of templates onto a single image frame for constructing the images used for crowdsourcing tasks is specified with six adjustable parameters: density, scale, color, transparency, rotation, and target object. Each of these parameters is described in the following paragraphs.

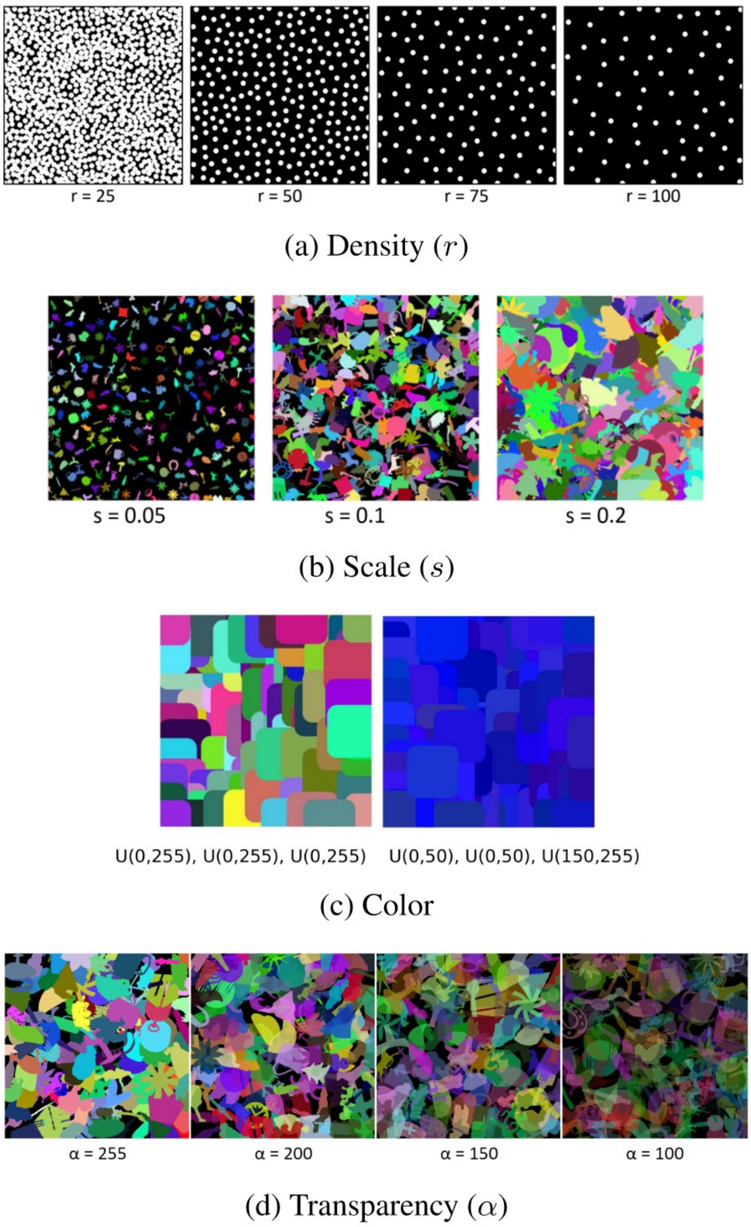

Figure S1: Example images generated by varying parameters

- **Density:** The center points of instantiated templates are set using a Poisson-disk distribution. This distribution produces closely packed individual points while maintaining a specified pixel distance, r. An example of the placement of dots for four increasing settings of r (by decreasing density) on a $1,000 \times 1,000$ pixel background is shown in Figure S1a. Once the dots are placed, the selected templates are inserted into the image to substitute them.
- **Scale:** The scale parameter, s, is defined on the interval $[0,1]$ and is randomly drawn from a triangular distribution $T(a,b,c)$. This parameter specifies the relative size of the templates within an image, i.e., the ratio of the height of the object/shape template to the background. Figure S1b displays three images generated by increasing the value of s; the larger s is, the more cluttered the generated image becomes.
- **Color:** The colors of the instantiated templates are set using RGB channels (red, green, and blue) where each channel is encoded as a number between 0 and 255; a specific combination of these values generates a unique color. Each instantiated object is assigned a single color. The RGB channel values are uniformly sampled from a restricted range, as specified by the distributions $U(a^R, b^R)$, $U(a^G, b^G)$, $U(a^B, b^B)$, where a and b are lower and upper bounds, respectively. Figure S1c displays an example of two color distributions. Alternatively, the color of an instantiated object can be randomly selected from a discrete set of RGB tuples.
- **Transparency:** The transparency value of a template, α, is drawn from a discrete uniform distribution $U(a,b)$. The parameter α is a number between 0 and 255, where smaller (larger, resp.) values are more transparent (opaque, resp.). Figure S1d displays four images generated with decreasing values of α.
- **Rotation:** The relative rotation, in degrees, of individual templates for an image is chosen by sampling from a uniform distribution $U(a,b)$.
- **Target Object:** When the image contains a specified target object, all templates of the same class are removed from the normal generation process. The target object template is used in place of a single random template to ensure it is present only once on the image frame.

2 EXPERIMENT IMAGES

This section will provide additional details on the images images used in the featured studies.

Images across all experiments were generated with a $1,080 \times 1,080$ pixel beige background (RGB values $(245, 245, 220)$). The rotation of all object templates follows the uniform distribution $U(0, 360)$. The remaining parameters are specific to each experiment. In experiment sets C and D we use images from four difficulty levels; "very difficult", "difficult", "average", and "easy", with densities 90, 100, 115, and 150 respectively. See Table S1.

3 TRADITIONAL VOTING METHODS

This section provides a brief overview of three traditional voting methods: Majority Voting, Confidence Weighted Majority Voting, and Surprisingly Popular Voting. Each of these methods uses a different input format and/or multiple inputs for the classification task.

- **Majority Voting (MV):** Majority Voting is the most widely used aggregation method due to its computational simplicity. Recall that in the featured experiments each participant's binary choice answer can be either 0 or 1. Therefore in this study, for an image i_k, the binary choice value that receives the highest number of votes is selected as the final label when MV is used. This label can be written as

$$y_k^* = \underset{l \in \{0,1\}}{\arg\max} \sum_{p_j \in P(i_k)} \mathbb{1}(l_k^j = l),$$

Sets C and D Examples	Positive	Negative
**Very Difficult**<br>Density: 90<br>Scale: $T(0.25, 0.35, 0.40)$<br>Color:<br>- $(31, 28, 28)$<br>- $(20, 92, 163)$<br>- $(89, 135, 28)$<br>- $(196, 130, 23)$<br>Transparency: $U(150, 200)$<br>Bat Location: Bottom, center	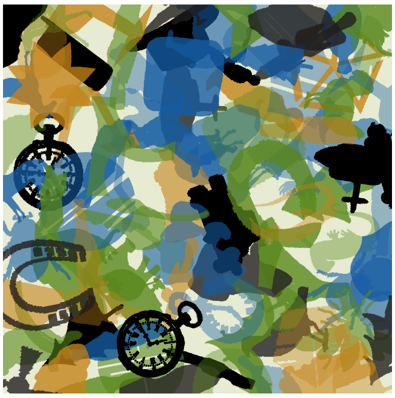	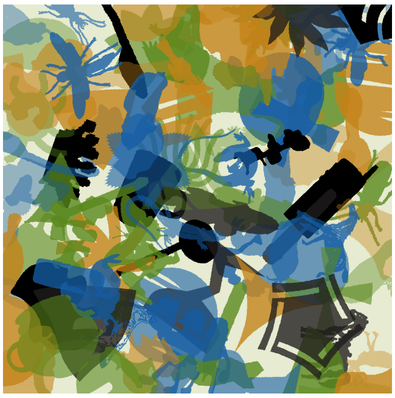
**Difficult**<br>Density: 100<br>Scale: $T(0.25, 0.35, 0.40)$<br>Color:<br>- $(31, 28, 28)$<br>- $(20, 92, 163)$<br>- $(89, 135, 28)$<br>- $(196, 130, 23)$<br>Transparency: $U(150, 200)$<br>Bat Location: Top, left	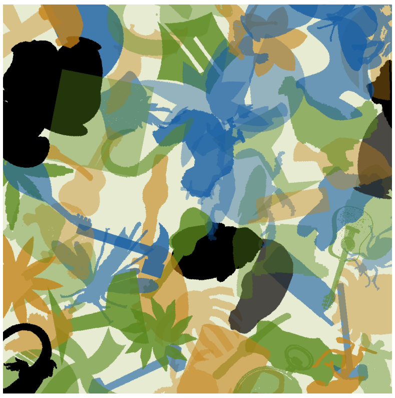	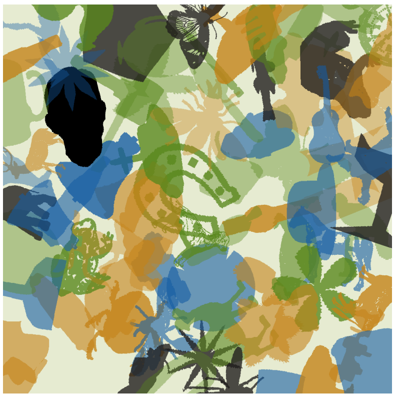
**Average**<br>Density: 115<br>Scale: $T(0.25, 0.35, 0.40)$<br>Color:<br>- $(31, 28, 28)$<br>- $(20, 92, 163)$<br>- $(89, 135, 28)$<br>- $(196, 130, 23)$<br>Transparency: $U(150, 200)$<br>Bat Location: Bottom, left	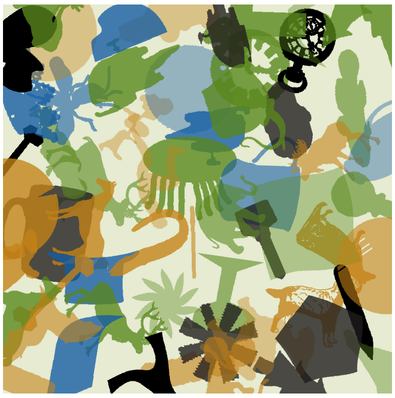	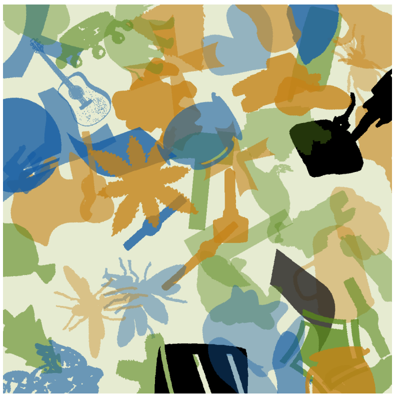
**Easy**<br>Density: 150<br>Scale: $T(0.25, 0.35, 0.40)$<br>Color:<br>- $(31, 28, 28)$<br>- $(20, 92, 163)$<br>- $(89, 135, 28)$<br>- $(196, 130, 23)$<br>Bat Location: Center	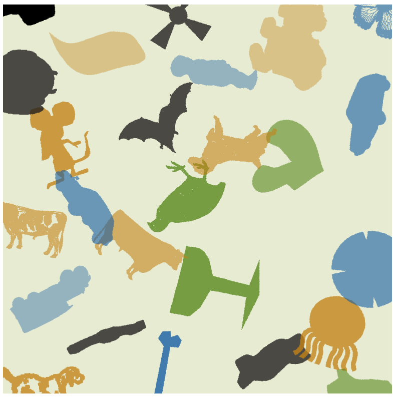	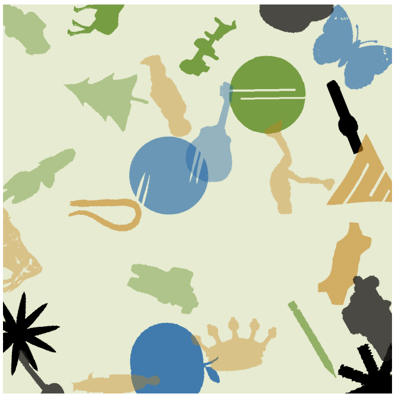

Table S1. Experiment Sets C and D sample images

where $\mathbb{1}(.)$ is an indicator function, which is equal to 1 whenever the given argument inside the bracket is true, and it is equal to 0 otherwise.

- **Confidence Weighted Majority Voting (CWMV):** An implicit assumption of Majority Voting is that participants within a crowdsourcing platform are equally reliable and, therefore, their provided labels should have equal weights in the aggregate outcome. This convention is not always ideal, specially in the presence of noisy and indecisive labelers. Previous research has found that participants can accurately assess their individual confidence in their independently formed decisions (e.g., see (Meyen et al., 2021)). We devise an intuitive aggregation approach that leverages this insight by weighing participants' labels according to their self-reported confidence values. More weight is given to participants who are more confident about their answers, and the final label of the image is chosen as the response whose summed confidence values is highest.

  In brief the predicted label of image i_k using this aggregation method can be written as

$$y_k^* = \underset{l \in \{0,1\}}{\arg\max} \sum_{p_j \in P(i_k)} c_k^{j*} \mathbb{1}(l_k^j = l).$$

- **Surprisingly Popular Voting (SPV):** The Surprisingly Popular Voting method leverages the idea that for some domain-specific questions where the majority of the crowd is highly inaccurate, participants who are accurate but are in the minority may also know that their response is rare (Rutchick et al., 2020). For a given question, the SPV method takes into account two groups of participants: Participants who agree on a label, and participants who think the given label will be the choice provided by the majority. The label that maximizes the difference between these two groups is selected as the final label. The predicted label of image i_k using this method can be written as

$$y_k^* = \underset{l \in \{0,1\}}{\arg\max} \sum_{p_j \in P(i_k)} \left[\mathbb{1}(l_k^j = l) - \mathbb{1}(g_k^j = l) \right].$$

4 DISTRIBUTION OF PARTICIPANTS' RESPONSE TIME

This section analyzes the relationship between the average response time of the participants (i.e., the time it took to complete the task) and the accuracy of their responses. The accuracy value of each participant, $acc_i \in \{0.0, 0.1, ..., 0.9, 1.0\}$ is calculated as the ratio of their correct responses over the total number of labeling tasks completed by the individual. To simplify the graphs, the accuracy values are rounded to one decimal place. Similar to Christoforou et al. (2021), the Empirical Cumulative Distribution Function (ECDF) of the different accuracy groups (i.e., set of participants with similar accuracy values) are plotted against the average response times (in Figure S2) to analyze this relationship for each Experiment Set. The ECDF is computed using the Kaplan-Meier estimate. Note that the graphs are constructed after removing the 35 participants identified as insincere using Criteria 2.

From Figure S2, it is clear that only a very small fraction of the participants took less than 10 seconds to complete the tasks, on an average, and that the accuracy of those participants was lower. Specifically, the three participants with an average response time of less than 10 seconds had an accuracy of 0.4 in Experiment Set A. Participants with high accuracy (> 0.6) took more than 20 seconds, on average, to complete each labeling task. This observation serves to justify the imposition of the 10-second rule in Criteria 1 for filtering insincere participants. Among the 321 participants used in this analysis, only two participants utilized the complete 60 seconds for each task (both for the imbalanced datasets); their accuracy value was relatively low (< 0.6).

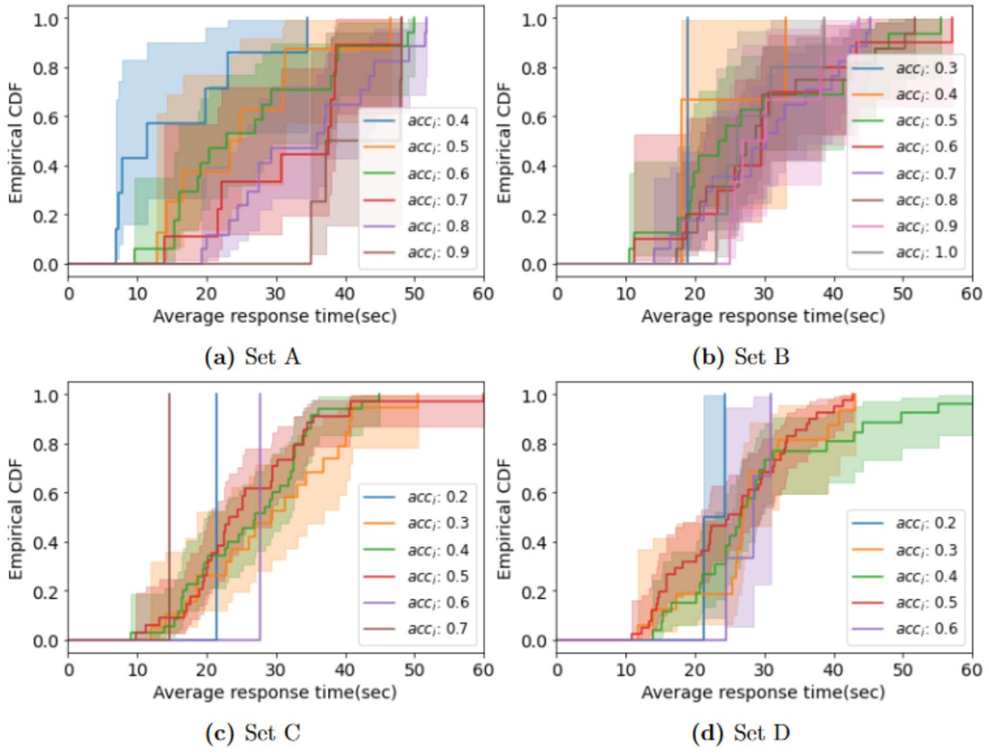

Figure S2: The Empirical CDF of the average response times

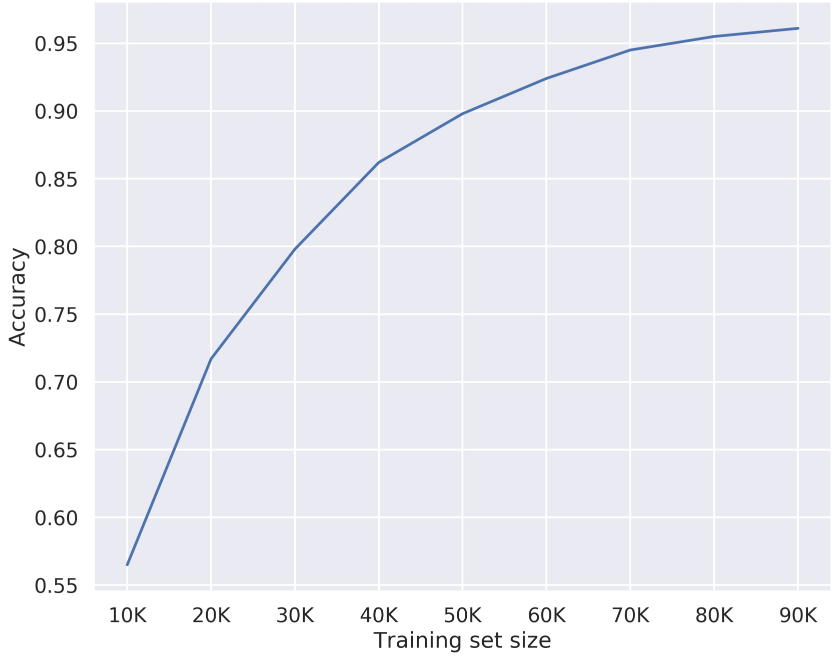

Figure S3: Validation accuracy vs. Training set size

5 RESNET-50 ARCHITECTURE AND PERFORMANCE

The original ResNet-50 architecture (He et al., 2015a) was trained on the ImageNet (Russakovsky et al., 2014) dataset, which consists of 1.28 million images and 1,000 different classes. We modified the fully connected layers of the standard architecture to make it compatible with the binary classification task. We used the Adam optimizer (Kingma and Ba, 2014) with default parameters (learning rate = 10^{-3}, $\beta_1 = 0.99$, $\beta_2 = 0.999$) and He's method (He et al., 2015b) to initialize the weights.

Due to length considerations, the figure showing accuracy as a function of the training set size for the automated ResNet-50 classifier are presented in the Supplementary Materials, and only the key points are summarized herein. The largest training set size of 90k samples led to more than 95% accuracy on the balanced validation set (consisting of 5k positive and 5k negative samples). However, when trained on the smallest training set of 10k samples, the model performed only slightly better than random guessing. This confirms that deep learning models almost always benefit from large datasets, given that the network has enough parameters to capture the learnable features.

Layer Name	Output Size	Layers
conv1	112×112	7×7, 64, stride 2
conv2_x	56×56	3×3 maxpool, stride 2
		$\begin{bmatrix} 1\times1, 64 \\ 3\times3, 64 \\ 1\times1, 256 \end{bmatrix} \times 3$
conv3_x	28×28	$\begin{bmatrix} 1\times1, 128 \\ 3\times3, 128 \\ 1\times1, 512 \end{bmatrix} \times 4$
conv4_x	14×14	$\begin{bmatrix} 1\times1, 256 \\ 3\times3, 256 \\ 1\times1, 1024 \end{bmatrix} \times 6$
conv5_x	7×7	$\begin{bmatrix} 1\times1, 512 \\ 3\times3, 512 \\ 1\times1, 2048 \end{bmatrix} \times 3$
fc1	1024×1	dropout, 2048-d fc, relu
fc2	256×1	dropout, 1024-d fc, relu
fc3	128×1	dropout, 256-d fc, relu
fc4	1×1	dropout, 128-d fc

Table S2. Modified version of the ResNet-50 architecture diagram

REFERENCES

Christoforou, E., Fernández Anta, A., and Sánchez, A. (2021). An experimental characterization of workers' behavior and accuracy in crowdsourced tasks. *Plos one* 16, e0252604

He, K., Zhang, X., Ren, S., and Sun, J. (2015a). Deep residual learning for image recognition. *arXiv preprint arXiv:1512.03385*

[Dataset] He, K., Zhang, X., Ren, S., and Sun, J. (2015b). Delving deep into rectifiers: Surpassing human-level performance on imagenet classification

Kingma, D. P. and Ba, J. (2014). Adam: A method for stochastic optimization. *arXiv preprint arXiv:1412.6980*

Meyen, S., Sigg, D. M., von Luxburg, U., and Franz, V. H. (2021). Group decisions based on confidence weighted majority voting. *Cognitive research: principles and implications* 6, 1–13

Russakovsky, O., Deng, J., Su, H., Krause, J., Satheesh, S., Ma, S., et al. (2014). Imagenet large scale visual recognition challenge. *CoRR* abs/1409.0575

Rutchick, A. M., Ross, B. J., Calvillo, D. P., and Mesick, C. C. (2020). Does the "surprisingly popular" method yield accurate crowdsourced predictions? *Cognitive research: principles and implications* 5, 1–10
